# Supplementary material for: Metal-organic framework derived crystalline nanocarbon for Fenton-like reaction
Source: Nat Commun. 2024 Jul 23;15:6199. doi: 10.1038/s41467-024-50476-w (PMC11266689; doi:10.1038/s41467-024-50476-w)
Supplement: Supplementary file 1 — Supplementary Information [file 41467_2024_50476_MOESM1_ESM.pdf]

Supplementary information

# Metal-organic framework derived crystalline nanocarbon for Fenton-like reaction

Tingting Lian[1], Li Xu[2], Diana Piankova[1], Jin-Lin Yang[3], Nadezda V. Tarakina[1], Yang Wang[1,2*], Markus Antonietti[1]

[1]Department of Colloid Chemistry, Max Planck Institute of Colloids and Interfaces, 14476 Potsdam, Germany.

[2]Department of Environmental Science and engineering, University of Science and Technology of China, 230026 Hefei, China.

[3]School of Physical and Mathematical Sciences, Nanyang Technological University, 637371 Singapore, Singapore.

*Corresponding author.

Email: ywangese@ustc.edu.cn

**This PDF file includes：**

Supplementary Notes 1-4

Supplementary Figures 1-31

Supplementary Tables 1-6

Supplementary References 1-73

**Supplementary Note 1**

Strategy for NaCl-assisted synthesis of carbon materials: recrystallization and salt reactor

Salt template for microstructure control and molten salt strategy for preparing carbon materials with higher surface area, have been widely developed for catalysis and energy storage applications.[1] In particular, the mild NaCl as a template is inherently stable and can be easily removed with water. Theoretically, the regular crystal shape of NaCl is convenient for the unfolded growth of the polymer and compounds during the assembly process. Because of the high stability of NaCl with melting point at ~800 $^{o}$C, it potentially could avoid the collapse of template and the aggregation of final products. The combined merits entitle NaCl a promising candidate salt to preserve the pristine morphology while finely regulate the nanostructure.

Several efforts have been made to introduce NaCl into the carbonization of MOFs. Dong *et al*. firstly synthesized ZIF-67 nanosheets using NaCl as template to confine the growth along the salt microcrystal plane in MeOH solvent in 2017.[2] This NaCl-template confined bottom-up strategy to construct ultrathin 2D MOFs highlight the pivotal role of NaCl in the synthesis of 2D materials. Later in 2018, Wang and coauthors developed a NaCl-assisted molten method, who also proposed the concept of salt reactor, in which NaCl not only has a confinement effect but also could activate the surface of ZIF-8 during the carbonation process, leading to formation of nanosheets on the carbon skeleton.[3] Zhao *et al*. studied the templating and the exfoliation effects of NaCl in preparation of web-like carbon network.[4] In 2021, Waterhouse and coauthors introduced the single-atom configuration $FeN_4$ hosted on the platform of molten NaCl-assisted microporous carbon.[5] The subsequent studies showed that the exfoliation of NaCl promoted the formation of nanosheets on the surface. These efforts however only recognized the templating and/or exfoliation effects of NaCl, the carbonized products are amorphous carbon without any crystalline nanostructure.

Salt recrystallization method can help precursor fully sealed inside the NaCl crystals. During the pyrolysis, the recrystallized NaCl could function as a closed salt reactor to facilitate the interactions between metals and binding linkers for phase transformation, which can potentially generate active sites when the carbonization temperature reaches the melting point of NaCl. Ding and coauthors applied this salt recrystallization method to fix the self-assembled polyaniline,[6] the final carbon showed higher degree of graphitization and preserved the original 3D framework, due to the protective layer of NaCl and the effect of molten salt. The thick NaCl shell prevents the MOF precursor from decomposition at a relatively low temperature. Therefore, salt reactor through the recrystallization process could be a powerful method to benefit the formation of finely designed nanocarbon.

**Supplementary Note 2**

Topological conversion via hydrolysis of ZIF-8

ZIFs are a subfamily of metal organic frameworks (MOFs), the similar metal-imidazole-metal bond angle with Si-O-Si in silicon zeolites makes ZIFs come with zeolite-type topology. Because of the similar crystalline frameworks as zeolites, ZIFs show higher thermal and chemical stability than other MOFs, making them suitable candidates for a wider range of applications. Among these, the polymorphs comprising zinc (II) and 2-methylimidazole are important branches, in which four polymorphs/pseudopolymorphs of ZIF have been identified, including cubic ZIF-8, katsenite (Zn), monoclinic ZIF-8 (diamondoid (Zn)) and ZIF-L.

Cubic ZIF-8 is the most representative member constructed by tetrahedral coordination of Zn cations to N in 2-methylimidazole(2-MIM) linkers. Cubic ZIF-8 has been widely developed due to its intriguing topology, it possesses a sodalite (SOD) topology with a large cage (size: 11.6 Å), which is accessible through a narrow six-ring pore (size: 3.4 Å)[7]. Due to its high thermal stability and unique pore size distributions, cubic ZIF-8 shows impressive performances in gas/liquid separation, sensing, and heterogeneous catalysis. Monoclinic ZIF-8 was discovered during an attempt to synthesize the cubic ZIF-8 through hydrothermal method,[8] which presents dense nonporous structure and typical 1D channel with chair-like hexagonal apertures along the *b* axis of this monoclinic cell.[9,10] Nevertheless, monoclinic ZIF-8 did not attract extensive attention for further developments in synthetic methods and applications.

The transformation from cubic ZIF-8 to monoclinic ZIF-8 is thermodynamically preferred because its Gibbs free energy is -4.6 kJ.[11] Although the relative strong Zn-N could permit the survival of ZIF-8 in humid atmosphere, ZIF-8 is not stable in aqueous solution due to the hydrolysis effect.[12] In this work, we developed a mild and easy strategy by hydrolyzing cubic ZIF-8 in aqueous NaCl solution, which successfully changed the topology of ZIF-8 changed from cubic into monoclinic one. The dense and compact structure of monoclinic phase provides a new path to prepare NPCs from alternative precursors.

We postulate that the hydrolysis in aqueous NaCl solution occurs through the enhanced attack of water molecules on Zn-N bonds and the protonation of methylimidazole MIM, leading to the formation of reorganized interlayer hydrogen bonds. This process disrupts the cage structure along the *b* axis, resulting in structural contraction and reorganization. Subsequent evaporation and drying processes further weaken the interlayer forces, leading to further contraction of MIM layer and the formation of a dense, non-porous monoclinic phase. This process keeps in line with the well-studied conversion between ZIF-L and ZIF-8.[13,14]

**Supplementary Note 3**

<u>Carbon materials for advanced oxidation process</u>

Carbon materials as heterogeneous catalysts carry the combined merits of high chemical stability and environmental friendliness, promising their deployment in water remediation through advanced oxidation process (AOP). The reduced graphene oxide (rGO) was firstly

reported to show high activity in peroxymonosulfate (PMS, $HSO_5^-$) activation[15], which was then also exemplified in various nanoporous carbons (NPCs), such as nanodiamonds[16,17] and carbon nanotubes[18]. It is widely accepted that radical and non-radical pathways dominate the PMS activation process, while the latter finds its primary role in the above-mentioned NPCs[19-21]. More specifically, the singlet oxygen ($^1O_2$) and mediated electron transfer process were identified as the main reactive pathways without the generation of radicals, which are otherwise rarely detected in metal-based active sites and exhibit excellent reactivity in complex aquatic surroundings. The catalytic activation of persulfates relies on the activation of the peroxide O-O bond and the efficient electron transfer between the peroxide and the catalyst, leading to the generation of reactive species. The selectivity from radicals to non-radical pathways are decided by the distinct activation behavior of the O-O bond in PMS. Direct electron transfer from the catalytic system to PMS will cause the cleavage of O-O bonds to generate radicals[22]. To a lesser extent, the elongation of O-O bonds (without cleavage), only, will lead to the formation of activated-PMS (PMS*) as intermediate to enhance the interaction between PMS and catalyst, this process is likely accompanied by electron transfer[23,24]. Then, the decomposition of PMS* through electron transfer process will eventually contribute to the generation of reactive species. In other words, a proper electron-transfer tendency with relatively strong adsorption of PMS molecules to form a reactive intermediate while avoid the formation of radicals, is the key for non-radical pathways.

In general, the adsorption of PMS molecules on carbons can be explained by two main mechanisms: chemisorption and physisorption. Chemisorption occurs through chemical bonding, facilitated by the presence of heteroatom functionalities on the carbon surface[25]. On the other hand, physisorption takes place through $\pi$-$\pi$ interactions and electrostatic attraction between PMS and the carbon surface[26]. Both mechanisms can simultaneously contribute to the adsorption process of PMS on carbons. Firstly, the heteroatom doping (N[18,24], O[17], B[27], S[28], P[20], *etc.*) method could be proposed to boost the catalytic performance by modifying the surface properties of catalysts to chemisorb the reactants and form surface intermediates. Among these, N-doped NPCs have been extensively explored for PMS activation, in which the graphitic N-assisted electron transfer[19] was demonstrated to largely promote the activation process in carbon and metal/carbon composite. Introducing N into the carbon lattice could reshape the $sp^2$-hybridized carbon configuration by altering the electronic structure, generating positively charged sites to improve the adsorption capability of PMS through the electrostatic attraction[29]. Then, charge transfer from the N atoms with higher electronegativity to PMS molecule via carbon network-N(-)-C(+)-$HSO_5$(-) structure is expected to generate reactive species. Being similar with N doping in carbon materials, the higher electronegativity of oxygen compared with carbon could possibly generate significant dipole moments[30], which could serve as active centers to trigger the primary activation of substrate.

The carbon configuration and dimension are important factors for the electron transfer process in carbocatalysis. The conjugated π system presented in graphitic carbon networks has the potential to facilitate the activation of aromatic rings in organic compounds through electrophilic attack from reactive oxidation species (ROS)[26,31], even in nonradical processes. Therefore, the findings in several studies suggest that the activity of $sp^2$ hybridized carbon atoms surpasses that of $sp^3$ hybridized ones[25,31]. Once captured on the surface, the metastable intermediates (PMS*) are capable of oxidizing organic contaminants (electron donors) by either abstracting electrons through the carbon lattice (acting as an electron shutter)[32] or engaging in inner-sphere interactions (oxidant-organic bonds)[33]. To boost this non-radical pathway, an electron tunnel for rapid charge transport is essential to accomplish the redox process. While in typical NPCs, the carbon network only succeeds in promoting the electron transfer process around active centers to generated reactive species. The amorphous materials with chaotic skeleton in NPCs would unfortunately cause the collisions of electrons at defective or charge-trapping sites. In this regard, the well-ordered pristine nanocarbons can serve better for efficient electron transfer process, but generally characterized by limited active sites. Therefore, the synergy between abundant active sites and intact electron channels are both highly important for Fenton-like reactions in AOP.

## Supplementary Note 4

<u>Activity of the single Zn atom catalysts for PMS activation</u>

Generally, the central metal sources in the reported single-atom catalysts for catalytic oxidation processes are transition metal atoms with partially occupied 3*d* orbitals (*e.g.*, Fe, Co, Cu, Mn)[24,34-36], which are conducive to electron transfer during the reactions. Comparatively, the fully occupied 3d orbitals of Zn (*i.e.*, $3d^{10}$ configuration) largely tethers the electron mobility and therefore renders $Zn^{2+}$ intrinsically inactive for the catalytic reactions. This is reflected in the recent report in Fenton-like reaction using Zn-based single-atom catalyst, which shows substantially lower activity in the catalytic degradation of various pollutants. For example, the rate constant for the degradation of acid orange 7 (AO7) was 0.136 $min^{-1}$ (degradation time: 30 min)[37], while it was determined to be 38.5 $min^{-1}$ (degradation time: 5 s) in our case (Fig. 4b in main text). Apart from the differences in performance, the catalytic mechanism of the reported single-atom zinc catalysts also differs from those observed in our work. Adjusting the ratio of nitrogen (N) to carbon (C) or oxygen (O) elements coordinated to a single Zn atom can extend the distribution of Zn 4*s* states near the Fermi level, this could result in stronger hybridization of Zn 4*s* with empty $O_2$ 2*p** states, thereby facilitating electron transfer in the subsequent protonation process of adsorbed $O_2$[38]. Several cases have attempted to activate zinc sites based on this principle, employing a dissolved oxygen activation mechanism to enhance the electron transfer of electron-rich pollutants to PMS with the $Zn\text{-}N_4$ configuration. In these studies, dissolved oxygen is reduced at the zinc site to produce superoxide radicals ($\bullet O_2^-$), which can participate in the degradation of pollutants[37,39,40]. Although $\bullet O_2^-$ from dissolved oxygen activation

is only a minor oxidation pathway for degradation, the intermediate formed by the Zn center binding to PMS is considered to be a more significant active species. This dissolved oxygen activation mechanism serves as corroborative evidence that the zinc site can be activated as an active site.

In fact, the "active center" $ZnN_4$ is more of a binding site for PMS molecules, forming a surface complex and obtaining electrons from the electron-rich area (intermediate) around the Zn site, following a typical electron transfer path. For this surface complex mechanism, the purpose of pollutant degradation depends on the rapid electron transfer process near the active center. Yu et al. directed the synthesis of single-atom zinc sites on the edge of carbon-based carriers, which accelerated the electron transfer process by strengthening the interaction between zinc sites and edge carbon structures, further improving the catalytic degradation kinetics[41]. Specifically, the single-atom zinc sites on the edge are modulated by the surrounding carbon structure, and the adsorption of $HSO_5^-$ on the $ZnN_4$-edge site is relatively weak. $HSO_5^-$ is more likely to be first decomposed into H and $SO_5$ intermediate species on the $ZnN_4$-edge. The carbon-based structure near the Zn sites exhibits synergistic effect on the decomposition products of PMS molecules. Then, the nearby electron-rich N site bonds the separated H atoms with dangling bonds and stabilizes $SO_5$. Subsequently, the intermediate species are rapidly transformed into active species, which then participate in the degradation of pollutants. This mechanism highlights the importance of the edge structure of zinc sites, ensuring that PMS molecules are quickly transformed and activated. Despite these advancements, MCC-950 in our work demonstrates a kinetic activity 15 times higher than the best Zn-$N_4$-edge-NC catalysts, showcasing its superior catalytic performance. However, unlike Co metal centers, the Zn centers do not cause PMS to decompose into typical free radicals. Instead, the activation and degradation process are controlled by efficient electron transfer, reinforcing the high catalytic activity of single-atom catalysts.

Regarding the active mechanism of this work, the EPR results excluded the formation of $\bullet O_2^-$, differing from the active species in previous studies (Figure 4e in main text). To further explore the effect of dissolved oxygen (DO) on the degradation process in the MCC-950/PMS system, we conducted PMS activation experiments after removing DO from the system. The results showed no attenuation in the degradation process (Supplementary Fig. 19), indicating that DO in the MCC-950/PMS system does not participate in degradation process and does not produce $\bullet O_2^-$.

As we explained in the main text regarding the ultrafast kinetic mechanism of MCC-950, the retention of more than 20% by mass ratio of heteroatom doping in MCC-950 induces nearby carbon atoms to exhibit electron-deficient properties, which can act as PMS capture sites. Additionally, the synergistic effect of the nearby structure ensures the rapid progress of the electron transfer pathway. This provides a reasonable explanation for the superior performance of MCC-950 compared to single-atom zinc catalysts. Furthermore, combined with the observed change in the interlayer spacing of MCC-950 after acid etching and SEM images which show

thin layer morphologies (Supplementary Fig. 20), it can be inferred that zinc serves as an important intercalation agent for stabilizing the MCC structure rather than being the main active site contributing to the catalytic activity. This structural stabilization by zinc, rather than direct participation in the catalytic process, highlights the distinct mechanism through which MCC-950 achieves its high performance, emphasizing the role of the carbon structure and heteroatom doping in facilitating rapid and efficient electron transfer for PMS activation.

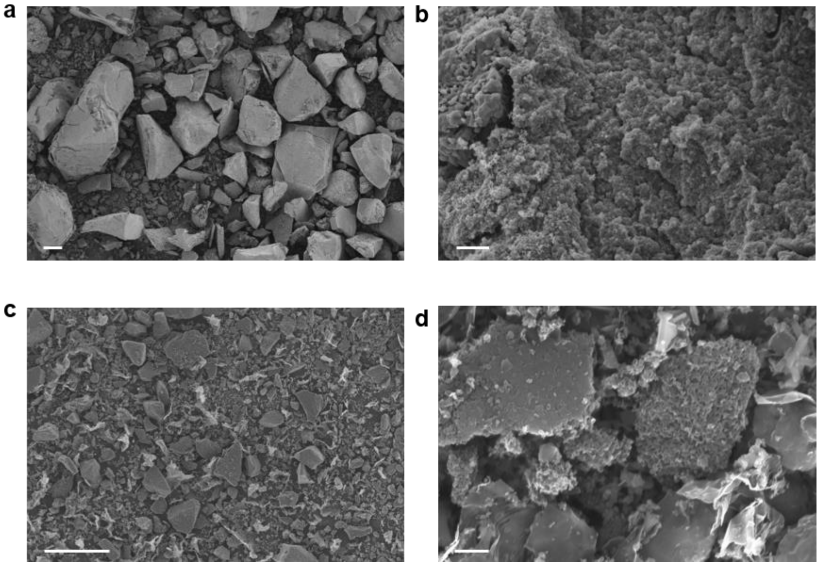


**Supplementary Fig. 1 | Microstructure of reference samples. a,b,** SEM images of traditional NPCs directly carbonized from ZIF-8 (scale bars in **a** and **b** are 100 μm and 2 μm, respectively). **c,d,** SEM images of NPCs prepared by carbonization of a mixture of ZIF-8 and NaCl (scale bars in **c** and **d** are 100 μm and 1 μm, respectively).

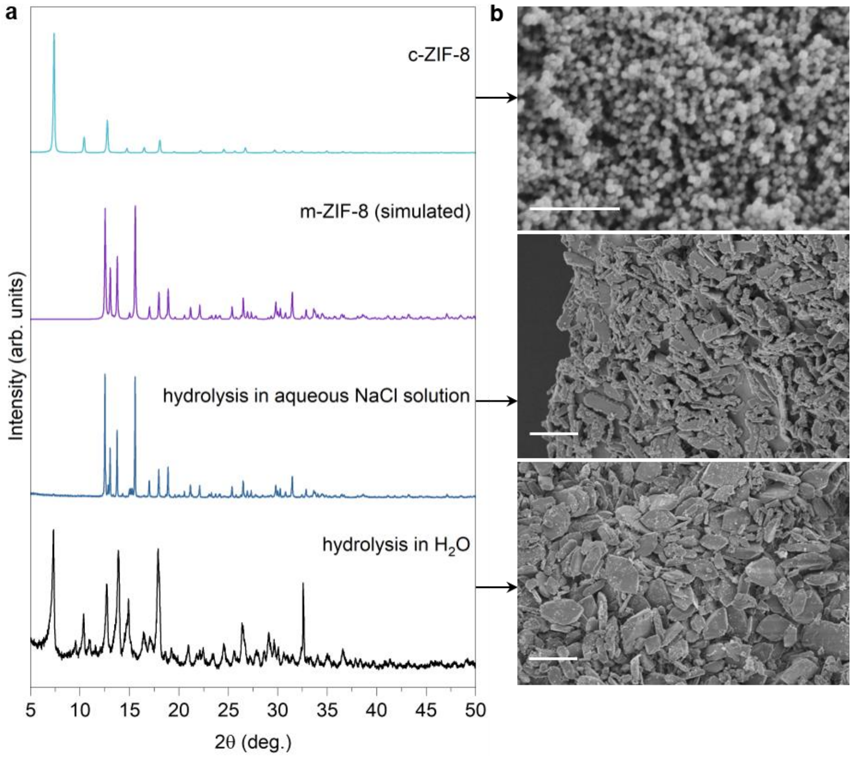


**Supplementary Fig. 2│Construction and characterization of ZIF-8 precursors under different treatments. a,** XRD patterns. **b,** SEM images. c-ZIF-8: cubic-ZIF-8, m-ZIF-8: monoclinic-ZIF-8. Scale bars of the upper, middle, and lower panels in **b** are 1 μm, 5 μm and 5 μm, respectively.

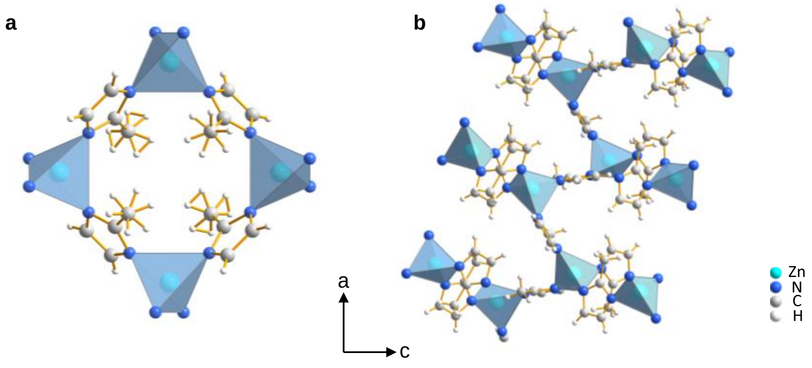


**Supplementary Fig. 3 | Crystal structures of ZIF-8. a,** c-ZIF-8 (nanoporous). **b,** m-ZIF-8 (dense).

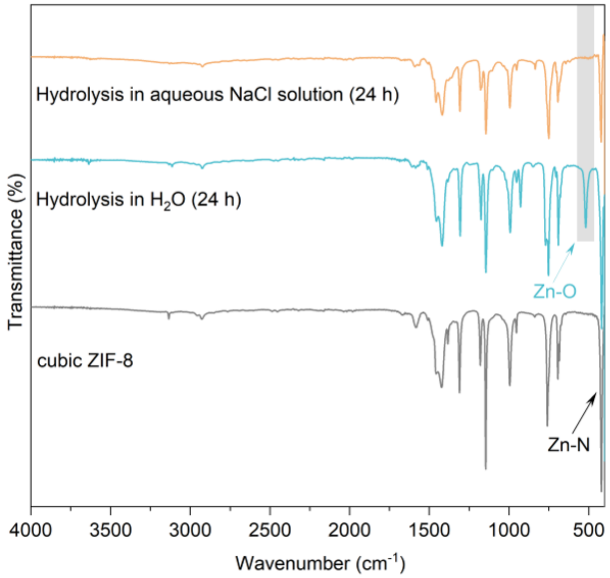


**Supplementary Fig. 4 | FT-IR spectra of ZIF-8 after hydrolysis.**

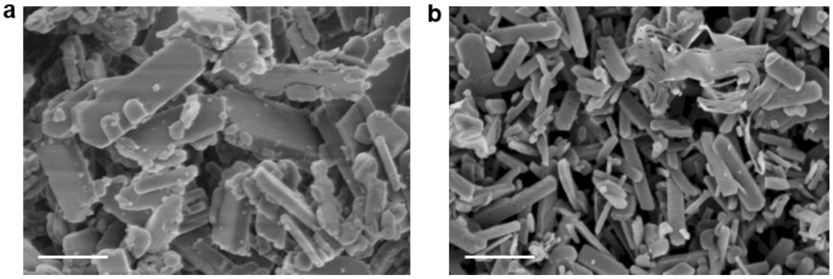


**Supplementary Fig. 5 | SEM images of ZIF-8 and carbonized product. a,** m-ZIF-8**. b,** MCC-950. Scale bars in **a** and **b** are 2 μm.

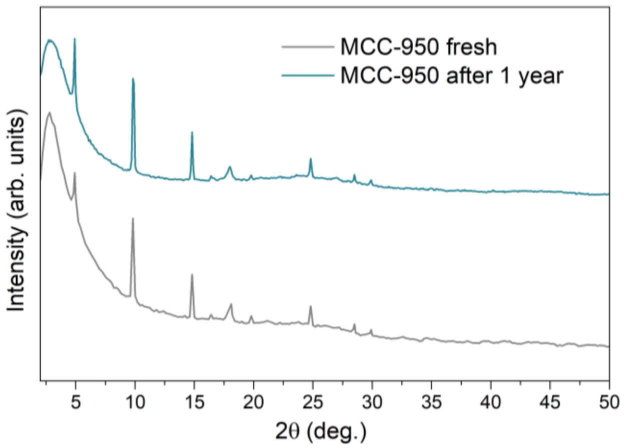


**Supplementary Fig. 6 | XRD patterns of fresh MCC-950 and the sample upon exposure in air atmosphere for one year.**

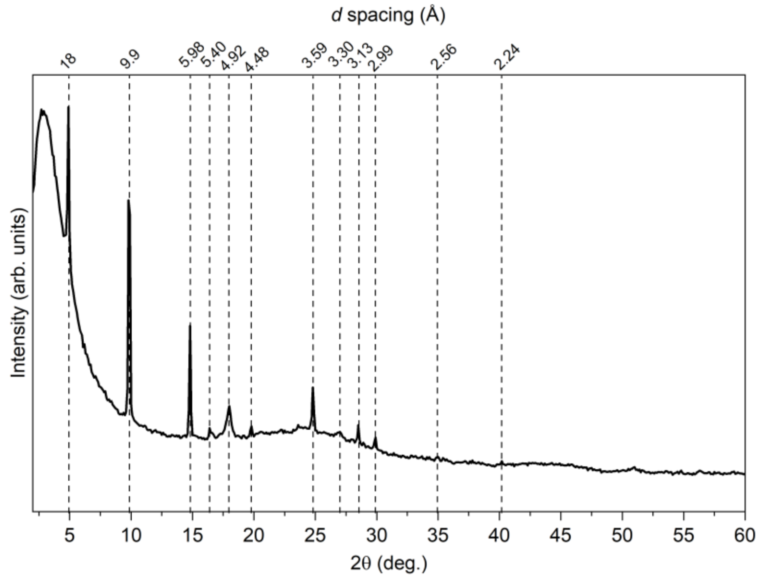


**Supplementary Fig. 7 | XRD pattern of MCC-950 with labelled interlayer spacing.**

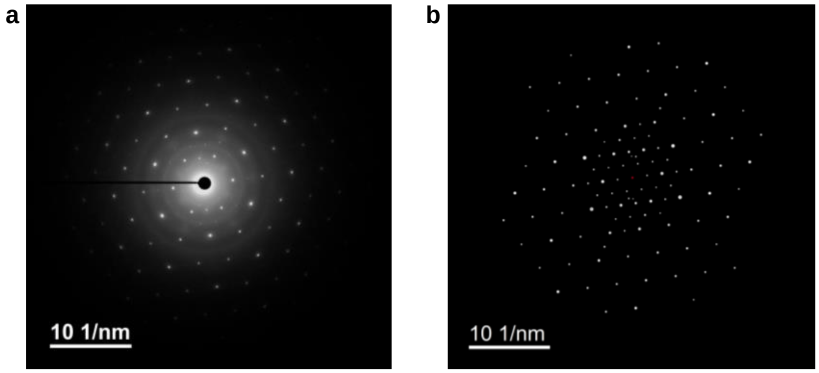


**Supplementary Fig. 8│SAED patterns of MCC-950. a,** Selected-area electron diffraction pattern. **b,** Labeled diffraction spots of MCC-950.

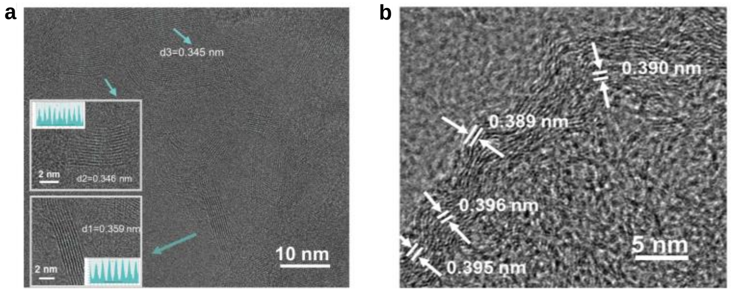


**Supplementary Fig. 9 | TEM images of MCC-950.**

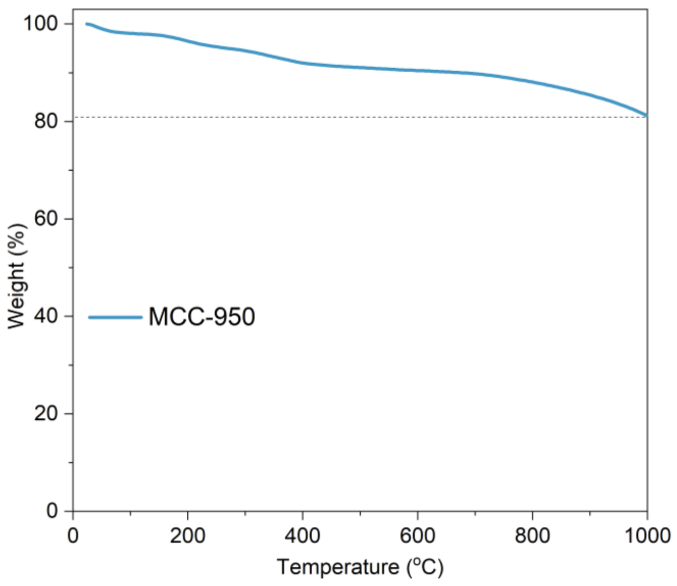


**Supplementary Fig. 10 | TGA curve of MCC-950.**

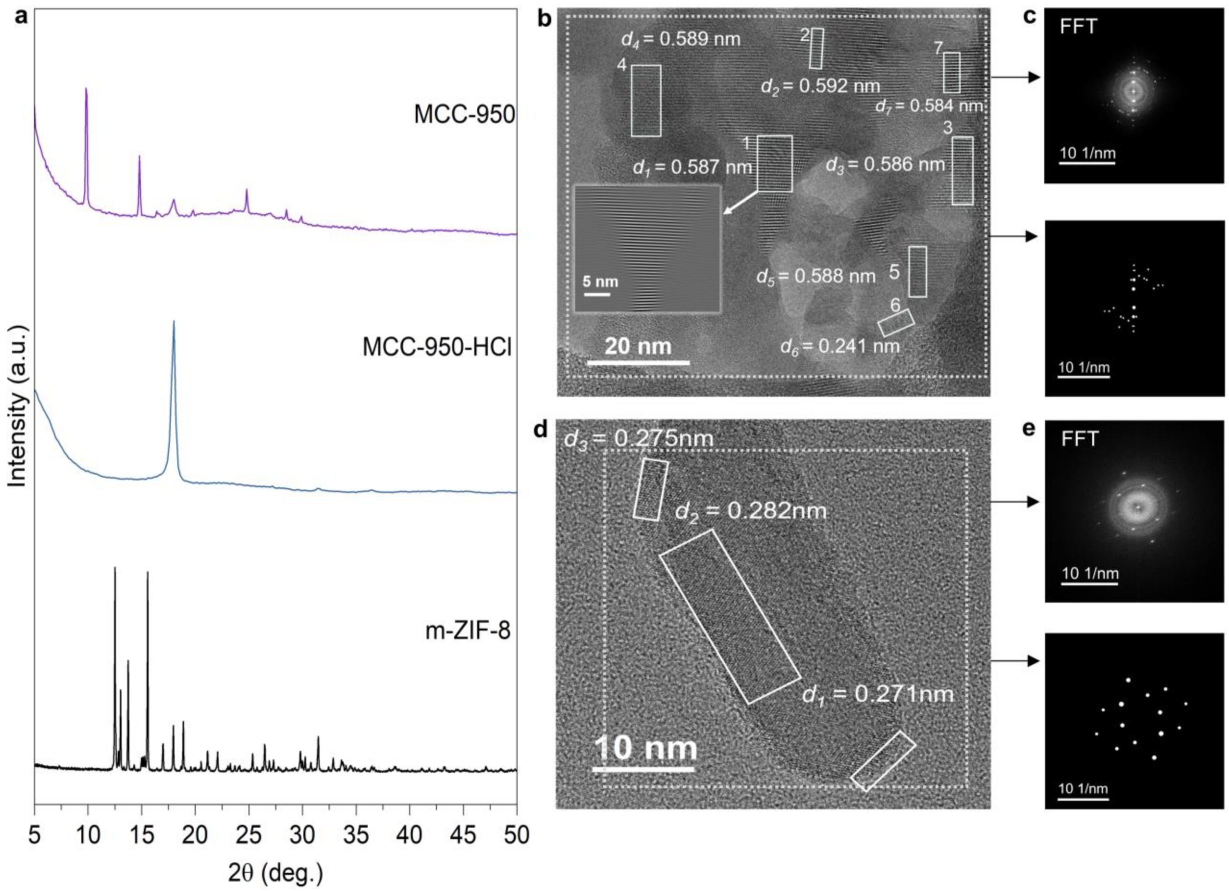


**Supplementary Fig. 11 | Characterizaitons of acid-leaching sample MCC-950-HCl. a,** XRD patterns of m-ZIF-8, MCC-950 and MCC-950-HCl. **b-e,** High-resolution TEM images (**b,d**), and corresponding fast Fourier transform (FFT) patterns (**c,e**) of MCC-950-HCl.

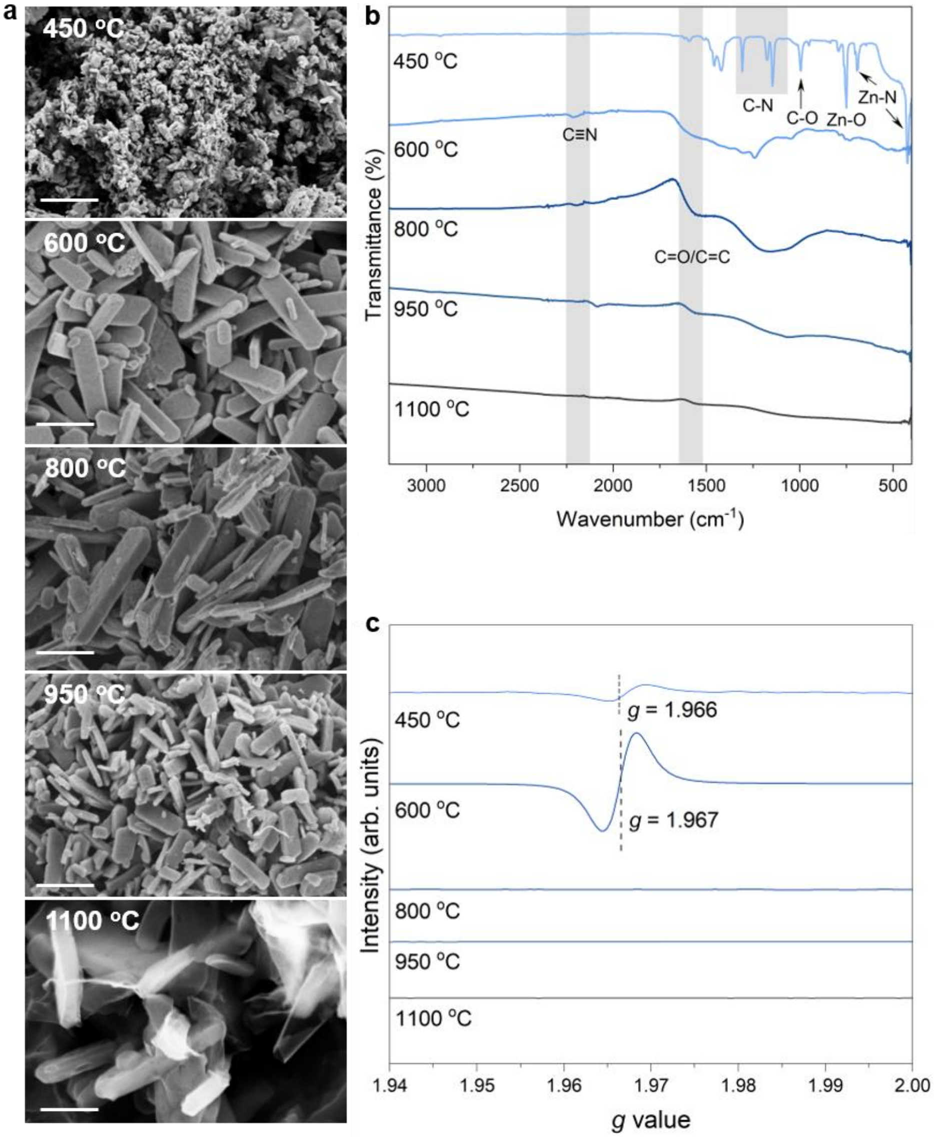


**Supplementary Fig. 12│Characterizations of the samples pyrolyzed at different temperatures. a,** SEM images. Scale bars: 2 μm for 450°C, 600°C, 800°C, 950°C and 1 μm for 1100 °C. **b,** FT-IR spectra. **c,** EPR spectra.

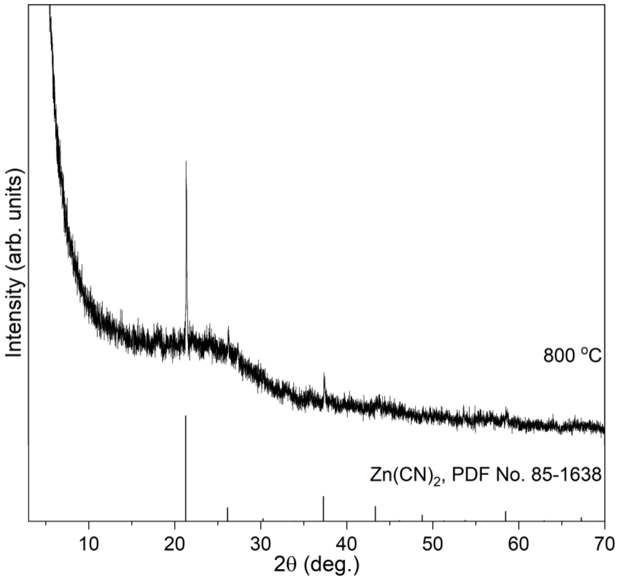


**Supplementary Fig. 13 │ XRD pattern of the sample pyrolyzed at 800 ºC.**

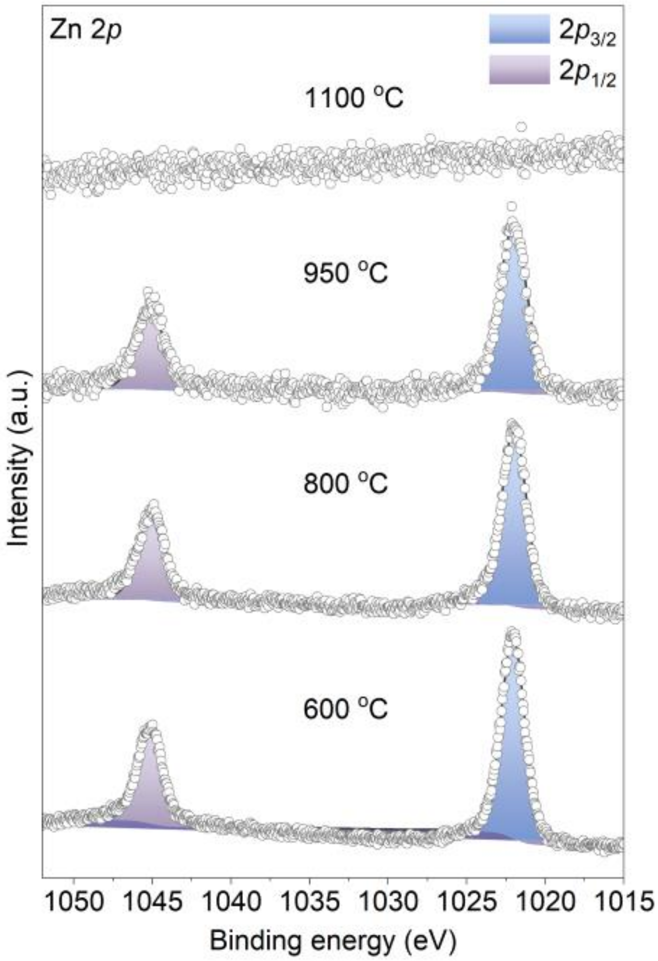


**Supplementary Fig. 14│Zn 2*p* XPS spectra of the samples pyrolyzed at different temperatures.** The two peaks centered at 1022 eV and 1045 eV point to the fact that Zn adopts “+2” valence state.

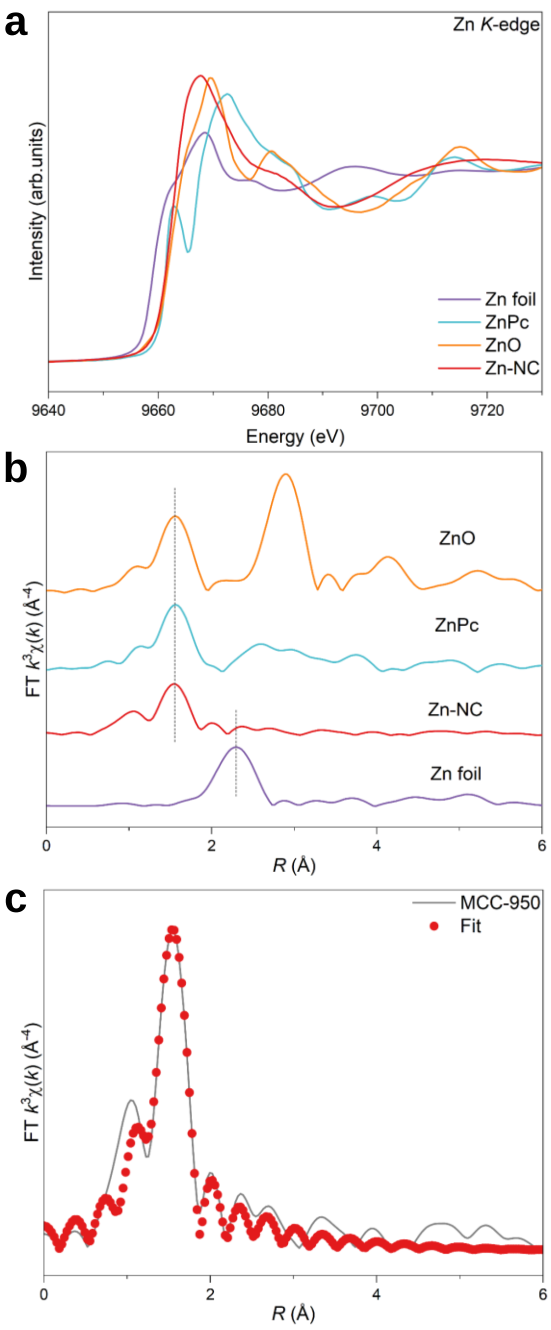


**Supplementary Fig. 15 | Chemical and coordination environment of Zn in MCC-950. a,** Zn K-edge XANES spectra of MCC-950 and reference samples (Zn foil, ZnO and ZnPc). **b,** FT $k^3$-weighted Zn K-edge EXAFS spectra of MCC-950 and reference samples. **c,** FT-EXAFS fitting curve of MCC-950 at Zn K-edge.

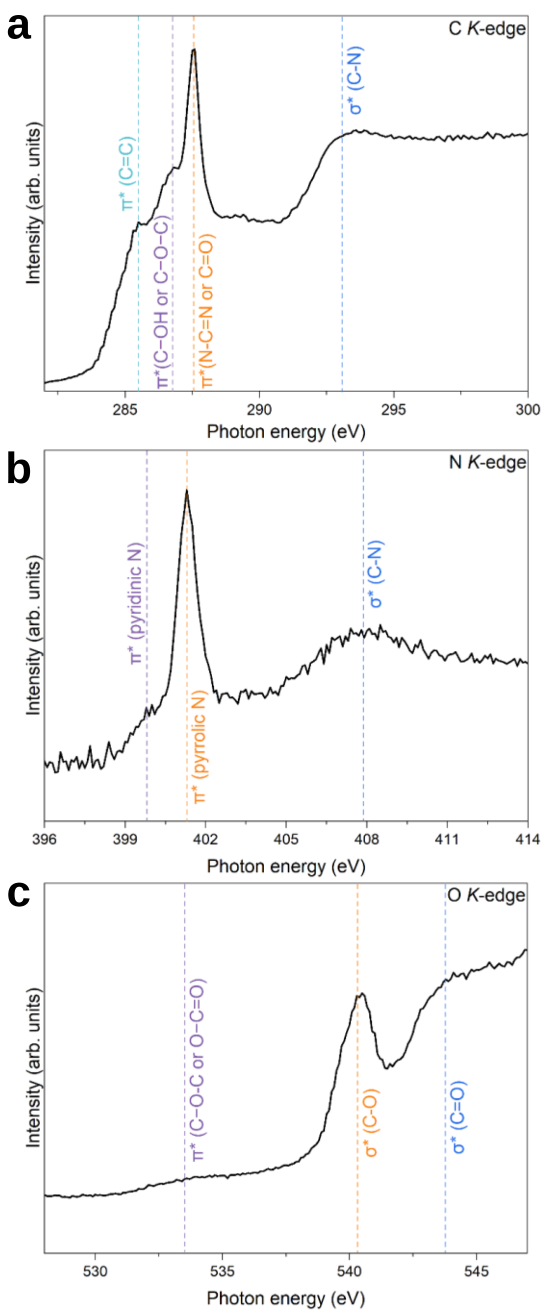


**Supplementary Fig. 16 | NEXAFS spectra of MCC-950. a,** C K-edge. **b,** N K-edge. **c,** O K-edge.

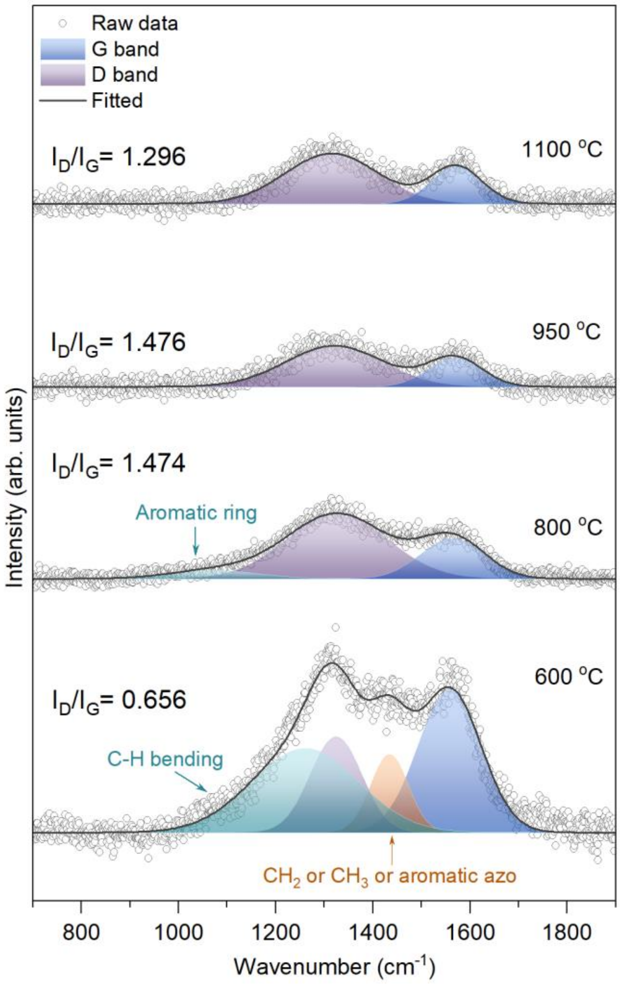


**Supplementary Fig. 17 │ Raman spectra of the samples pyrolyzed at different temperatures.**

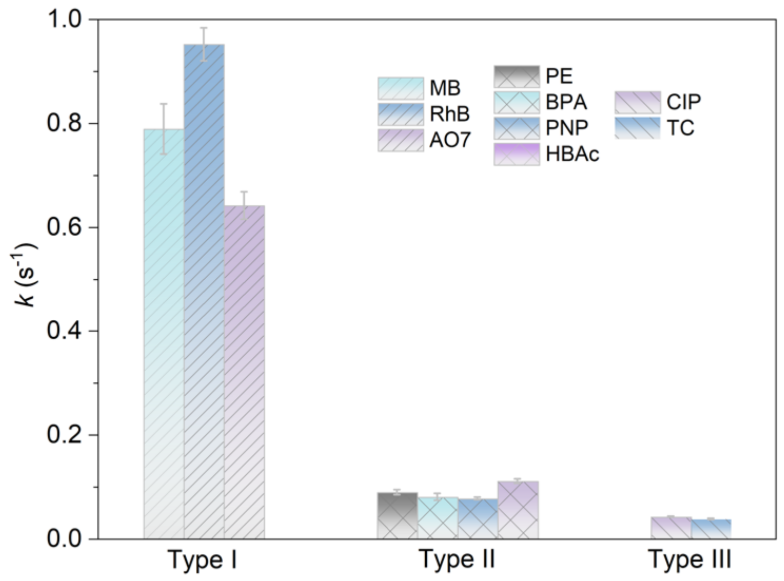


**Supplementary Fig. 18 | Kinetics values of pollutant degradation profiles in MCC-950/PMS system.** Error bars in represent the standard deviations of three independent measurements.

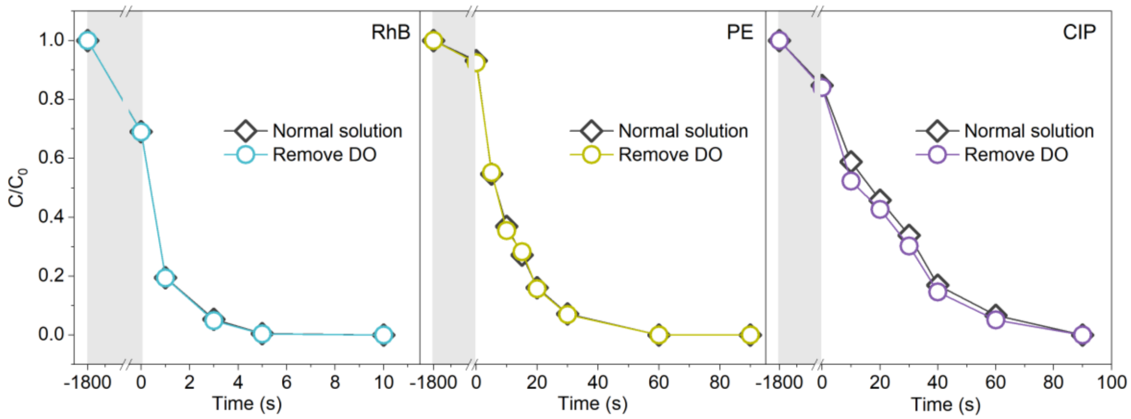


**Supplementary Fig. 19 | Degradation profiles of MCC-950 towards RhB, PE and CIP upon PMS activation in normal solution (with DO) and DO-removed solution.**

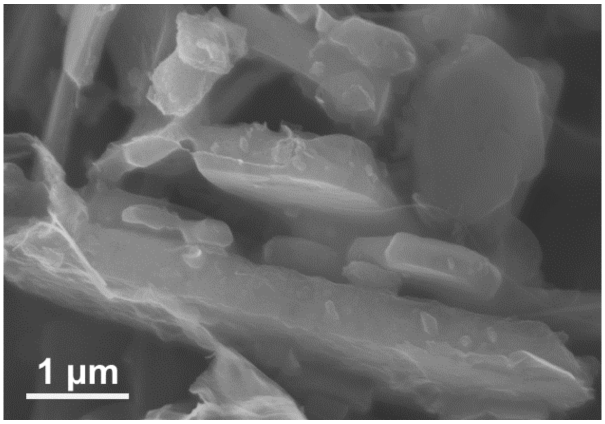


**Supplementary Fig. 20 | The SRM image of MCC-950 after acid etching by 1M HCl (sediment).**

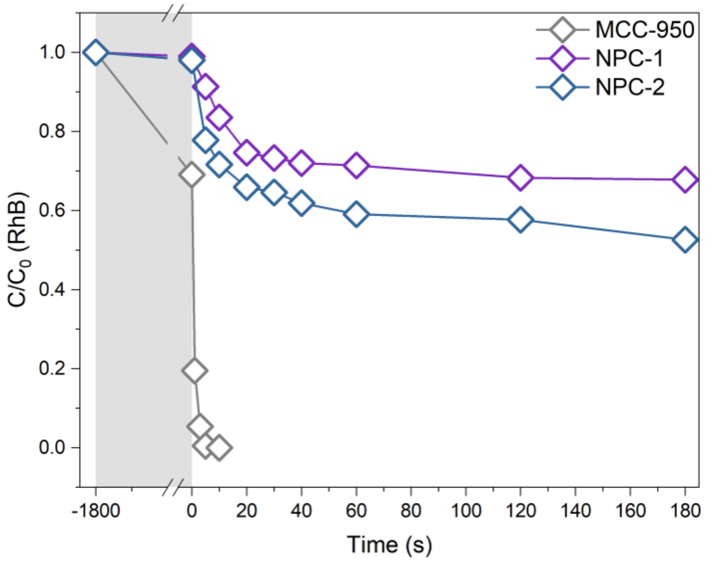


**Supplementary Fig. 21 | RhB degradation profiles in different carbon/PMS systems.** NPC-1 denotes the traditional NPC prepared by direct carbonization of cubic ZIF-8, while NPC-2 denotes the carbon product prepared by pyrolyzing the mixture of cubic ZIF-8 and NaCl ("Methods" section in main text).

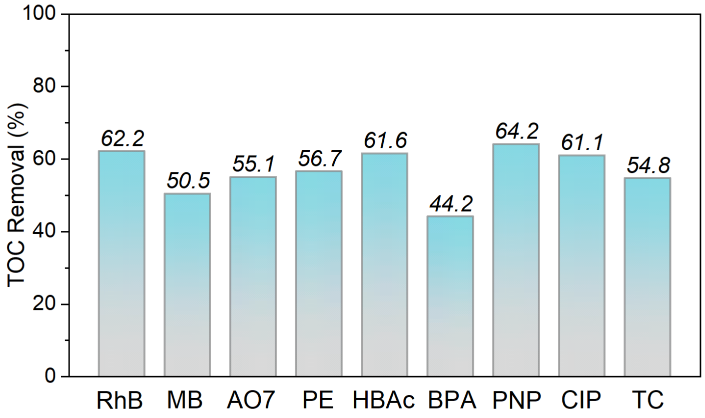


**Supplementary Fig. 22 | TOC removal efficiency of all 9 pollutants in this work.** Reaction condition: [pollutants] = 20 mg $L^{-1}$, [PMS] = 0.4 g $L^{-1}$, [catalyst] = 0.08 g $L^{-1}$, T = 298K, initial solution pH = 6.5, reaction duration: 2 min.

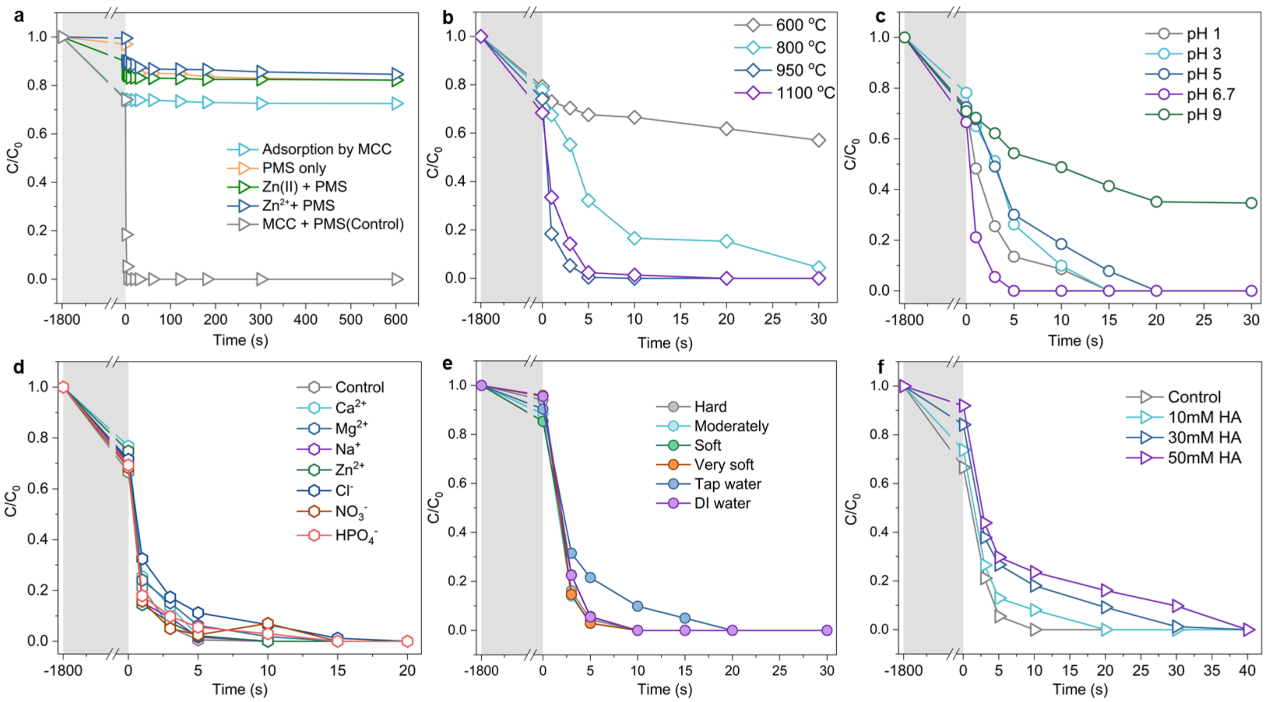


**Supplementary Fig. 23 | RhB degradation profiles in different systems. a,** Blank systems. Zn(II) was added in the form of $ZnSO_4$ solution while $Zn^{2+}$ was added in the form of ZnO solid. **b,** the samples pyrolyzed at different temperatures. **c,** Different initial pH values. **d,** Coexistence of different ions. **e,** Hardness. **f,** Humic acid. The marked regions (light grey shadow) represent the adsorption period (30 min) prior to catalytic reaction. Reaction condition: [pollutants] = 20 mg $L^{-1}$, [PMS] = 0.4 g $L^{-1}$, [catalyst] = 0.08 g $L^{-1}$, T = 298K.

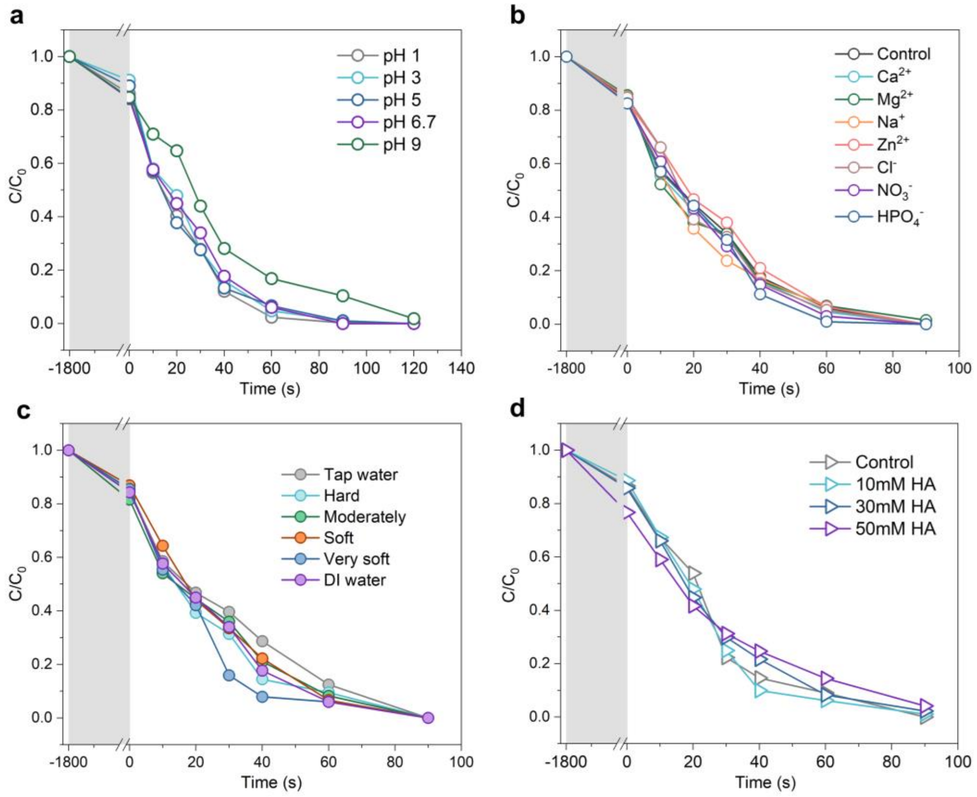


**Supplementary Fig. 24│CIP degradation profiles in different systems. a,** Different initial pH values. **b,** Coexistence of different ions. **c,** Hardness. **d,** Humic acid. The marked regions (light grey shadow) represent the adsorption period (30 min) prior to catalytic reaction. Reaction condition: [pollutants] = 20 mg $L^{-1}$, [PMS] = 0.4 g $L^{-1}$, [catalyst] = 0.08 g $L^{-1}$, T = 298K.

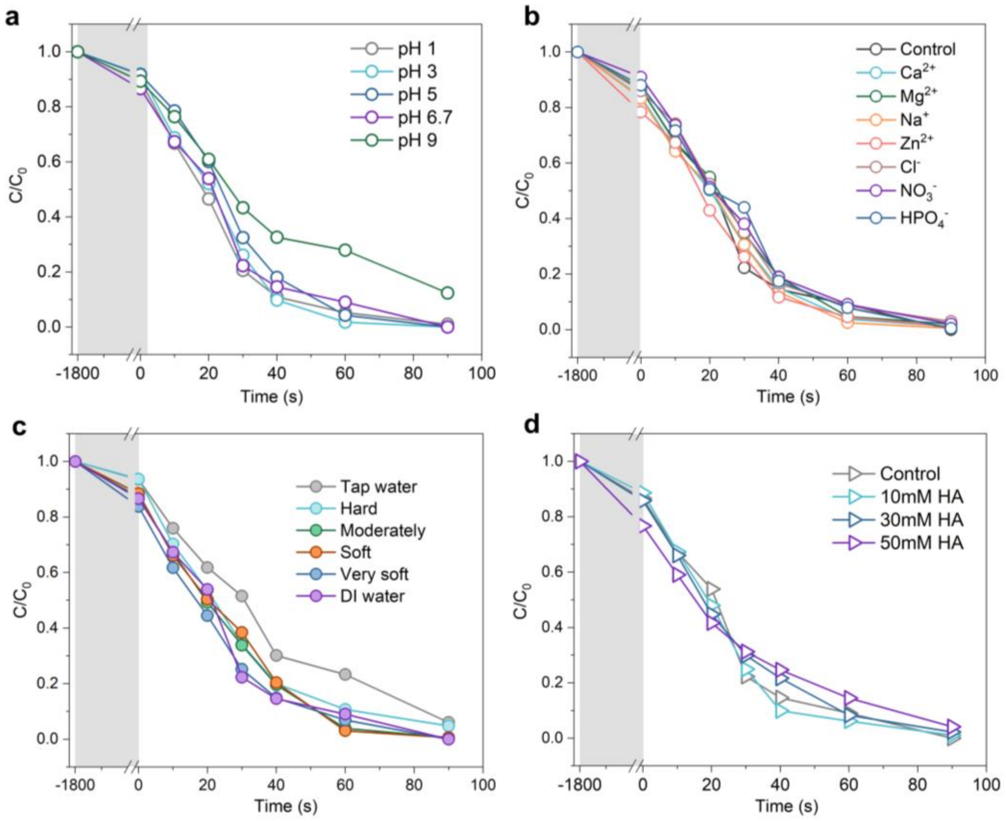


**Supplementary Fig. 25 | TC degradation profiles in different systems. a,** Different initial pH values. **b,** Coexistence of different ions. **c,** Hardness. **d,** Humic acid. The marked regions (light grey shadow) represent the adsorption period (30 min) prior to catalytic reaction. Reaction condition: [pollutants] = 20 mg $L^{-1}$, [PMS] = 0.4 g $L^{-1}$, [catalyst] = 0.08 g $L^{-1}$, T = 298K.

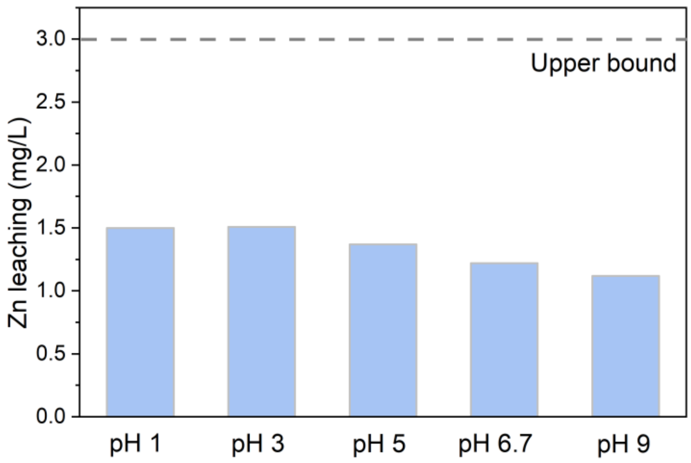


**Supplementary Fig. 26 | Zn leaching amount of MCC-950 with different initial pH values (pH=1, 3, 5, 6.7, 9).** Reaction condition: [pollutants] = 20 mg $L^{-1}$, [PMS] = 0.4 g $L^{-1}$, [catalyst] = 0.08 g $L^{-1}$, T = 298 K.

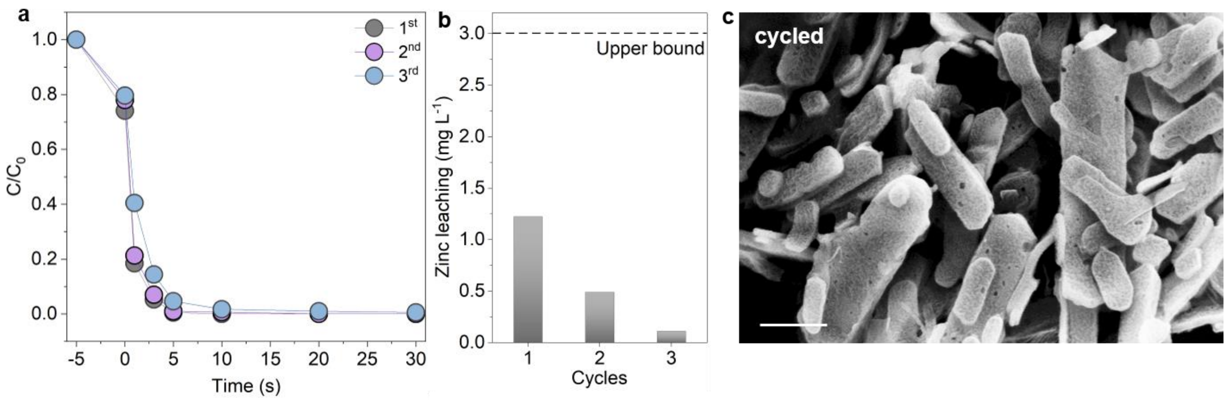


**Supplementary Fig. 27 | Durability of MCC-950**. **a,** Cycling experiments for RhB degradation using MCC-950. **b,** Zn leaching amount after each cycle. **c,** SEM image of MCC-950 after cycling experiment, scale bars: 1 μm. Reaction condition: [pollutants] = 20 mg $L^{-1}$, [PMS] = 0.4 g $L^{-1}$, [catalyst] = 0.08 g $L^{-1}$, T = 298K, initial solution pH = 6.5.

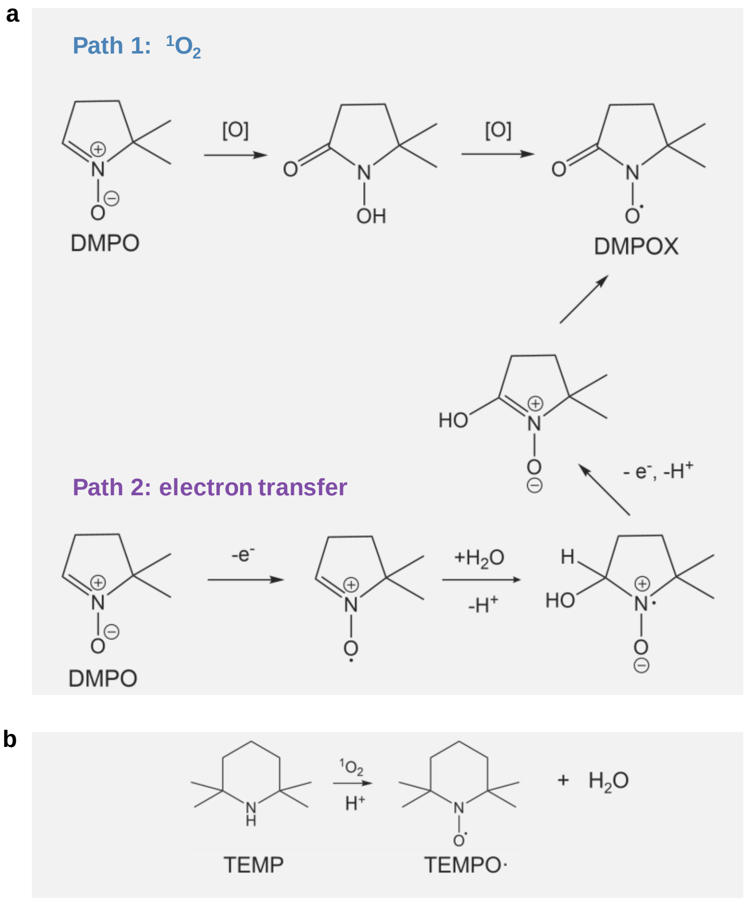


**Supplementary Fig. 28 | Reaction mechanism in EPR trapping experiments. a,** The formation of DMPOX. **b,** The formation of TEMPO.

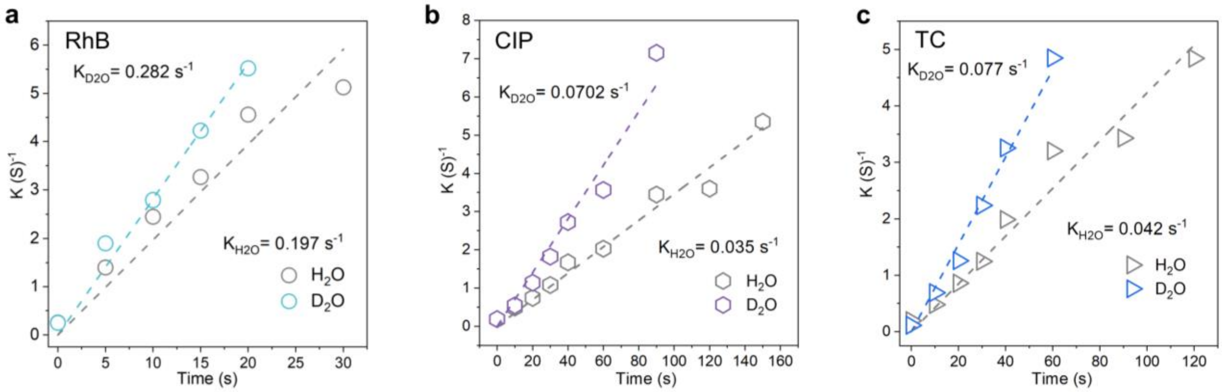


**Supplementary Fig. 29│Comparious kinetics of degradation in $H_2O$ and $D_2O$ for (a) RhB, (b) CIP and (c) TC.** Reaction condition: [pollutants] = 20 mg $L^{-1}$, [PMS] = 0.4 g $L^{-1}$, [catalyst] = 0.06 g $L^{-1}$, T = 298K, initial solution pH = 6.5.

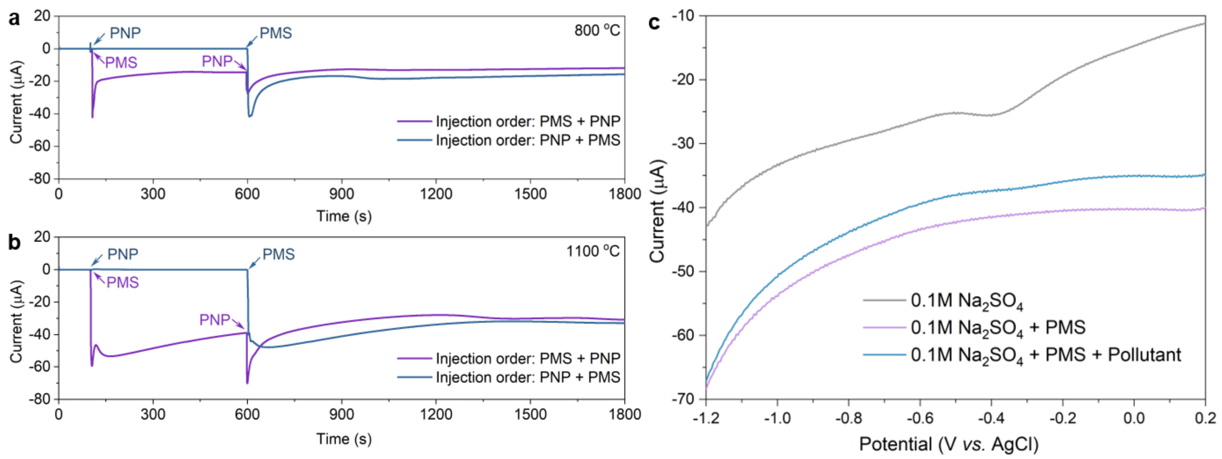


**Supplementary Fig. 30 | a,b,** Current responses after the sequential injection of PMS and PNP in the samples pyrolyzed at 800 °C (**a**) and 1100 °C (**b**). **c,** Linear seep voltammetry curves in different electrolytes.

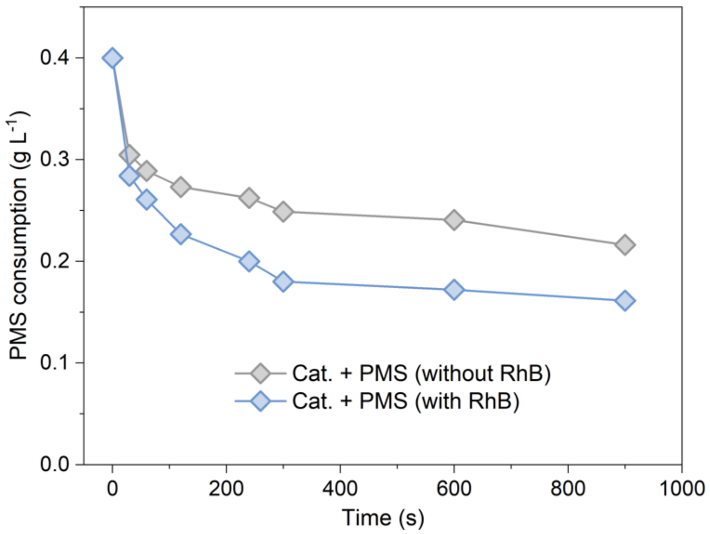


**Supplementary Fig. 31 | PMS consumption in MCC-950/PMS system with (12 mg) or without (9.5 mg) pollutant (RhB).** The initial amount of PMS is 20 mg.

**Supplementary Table 1 | Summary of MOF-derived carbons in various applications.**

| Year | precursor | Material | Method | Feature | Application | Reference | Note | |
|---|---|---|---|---|---|---|---|---|
| 2008 | MOF-5 | NPC | FA polymerized in the pores of MOF-5 and carbonized at 1000$^{o}$C for 8 h in Ar | No specific morphology, Amorphous | $H_2$ adsorption and electrochemical double-layered capacitor | Liu *et al.*[42] | first paper: MOF derived carbon | First-generation MOF-derived NPCs |
| 2011 | ZIF-8 | C1000 | FA/ZIF-8 composite pyrolyzed at different temperatures in Ar | No specific morphology, Amorphous | Supercapacitor | Jiang *et al.*[43] | | |
| 2012 | Al-PCP | NPC | Direct carbonization of Al-PCP | Fibrous shape, amorphous | Sensing | Hu *et al.*[44] | first paper: MOF direct carbonization | |
| 2012 | IRMOF-1 IRMOF-3 IRMOF-8 | MDCs | Direct carbonization in $N_2$ | Porous, amorphous | $H_2$ storage | Yang *et al.*[45] | | |
| 2012 | ZIF-8 | Z-900 | Direct carbonization | Reunited polyhedron, Amorphous | electrochemical capacitances | Chaikittisilp *et al.*[46] | | |
| 2015 | ZIF-8@ZIF-67 | NC@GC | Direct carbonization at 800 $^{o}$C for 3 h | Core-shell structure, amorphous | Electrochemical Supercapacitors | Tang *et al.*[47] | | Second-generation MOF-derived NPCs (Morphology preserved transformation and developing new morphologies) |
| 2016 | MOF-74-rod | CNRod, GNRib | Direct carbonization at 1000 $^{o}$C in Ar | Rod-shaped morphology, graphene nanoribbons, | Supercapacitors | Pachfule *et al.*[48] | | |
| 2017 | ZIF-8 | HMCNCs | m-$SiO_2$ modified ZIF-8 pyrolyzed at 900 in $N_2$ | Cubic morphology, hollow cavity, amorphous | Li-$SeS_2$ batteries | Liu *et al.*[49] | | |
| 2017 | ZIF-8 | HPCNFs-N | embedded (ZIF-8) nanoparticles into electrospun polyacrylonitrile (PAN) and then direct carbonization | Nanofiber composed with N-doped carbon, hollow nanoparticles, amorphous | Supercapacitors | Chen *et al.*[50] | | |
| 2017 | ZIF-67 | Co, N-C NS | NaCl-template confined ZIF-67 directly carbonized in $N_2$ | Nanosheet, amorphous | $O_2$ reduction reaction | Huang *et al.*[2] | | |

| | | | | | | | | |
|---|---|---|---|---|---|---|---|---|
| 2018 | ZIF-8 | Fe-SAs/NpS-HC | Direct carbonization of ZIF-8/Fe@PZS in Ar | Cubic morphology, amorphous | $O_2$ reduction reaction | Chen *et al.*[51] | first single-atom-anchored MOF-derived Carbon | New methods and hybrid materials development |
| 2021 | ZIF-8 | OCCs | $NaH_2PO_2$ assisted carbonization of ZIF-8 | Open carbon cages on cubic carbon | Aqueous Zn-ion hybrid supercapacitors | Hou *et al.*[52] | Gas-steamed MOF route | |
| 2021 | $NH_2$-MIL-125 | $TiO_2$@COF-x | Shell motif hybridization of COFs on MOFs, carbonized in $N_2$ | Carbonaceous particles embedded in core shell carbon structure | Capacitive deionization | Liu *et al.*[53] | MOF@COF for carbon | |

2

**Supplementary Table 2 │ Summary of *d*-distances corresponding to XRD patterns and HR-TEM results.**

| XRD patterns | | HR-TEM | | | |
|---|---|---|---|---|---|
| 2-Theta (°) | d (nm) | Diffraction spot number | d ($nm^{-1}$) | d' (nm) | Lattice d (nm) |
| **4.9** | **1.800** | D1 | 21.80-21.90 | 0.0456-0.0459 | **0.298** |
| **9.8** | **0.990** | D2 | 18.20-18.30 | 0.0546-0.0550 | **0.326** |
| **14.8** | **0.598** | D3 | 14.50-14.70 | 0.068-0.069 | **0.331** |
| **16.4** | **0.540** | D4 | 10.9-11.05 | 0.090-0.092 | 0.345 |
| **18** | **0.492** | D5 | 7.26-7.36 | 0.135-0.138 | 0.346 |
| 19.8 | 0.448 | D6 | 3.55-3.75 | **0.267-0.282** | 0.359 |
| 20.6 | 0.431 | D7 | 1.81-1.85 | **0.540-0.552** | |
| 22.2 | 0.399 | D8 | 2.61-2.66 | **0.375-0.383** | |
| 23.6 | 0.376 | D9 | 4.70-4.90 | 0.204-0.213 | |
| 24.8 | 0.359 | D10 | 6.60-6.70 | 0.149-0.152 | |
| **27** | **0.330** | D11 | 3.12-3.18 | **0.313-0.320** | |
| **28.5** | **0.313** | D12 | 6.14-6.46 | 0.154-0.163 | |
| **29.9** | **0.299** | D13 | 8.33-8.43 | 0.118-0.120 | |
| **40.2** | **0.224** | D14 | 9.60-9.70 | 0.103-0.104 | |
| | | D15 | 12.65-12.77 | 0.078-0.079 | |
| | | D16 | 15.80-15.95 | 0.0627-0.0633 | |
| | | D17 | 19.1-19.3 | 0.0518-0.0523 | |

**Supplementary Table 3 | Summary of physical properties and chemical composition of the samples pyrolyzed at different temperatures.**

| Materials | Element analysis (wt%) | | | ICP (wt%) | | |
|---|---|---|---|---|---|---|
| | C | N | O | Zn | $S_{BET}$ ($m^2 g^{-1}$) | $V_T$ ($m^3 g^{-1}$) |
| 600 $^oC$ | 40.25 | 29.84 | 1.41 | 28.5 | 152 | 0.25 |
| 800 $^oC$ | 58.12 | 21.36 | 6.72 | 13.8 | 1083 | 0.51 |
| 950 $^oC$ | 71.20 | 14.16 | 9.60 | 5.04 | 890 | 0.64 |
| 1100 $^oC$ | 76.48 | 13.02 | 10.23 | 0.27 | 2665 | 1.64 |

**Supplementary Table 4 | EXAFS fitting parameters at the Zn K-edge for MCC-950.**

| Sample | Path | CN | R (Å) | $\sigma_2$ ($10^{-3}$) | $\Delta E_0$ (eV) | R-factor |
|---|---|---|---|---|---|---|
| MCC-950 | Zn-N | 4.2 | 2.03 | 0.008 | 0.98 | 0.009 |

CN: coordination number; R: bond distance; $\sigma^2$: Debye-Waller factors; $\Delta E_0$: energy shift; R-factor: goodness of fit.

**Supplementary Table 5 | The physicochemical properties of pollutants used in this work.**

| Pollutant | Classification | Chemical Structure | Molecular weight (g mol$^{-1}$) | Solubility in water | Log Kow | pKa$_1$ | Toxicity ($LD_{50}$) | Nature |
|---|---|---|---|---|---|---|---|---|
| Methyl blue (MB) | | 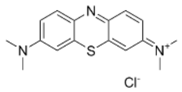 | 319.85 | 43.6 g L$^{-1}$ (25 ºC) | 0.75 | 3.14 | 1180 mg kg$^{-1}$ (Rat, oral) | Positive |
| Rhodamine B (RhB) | Dyes | 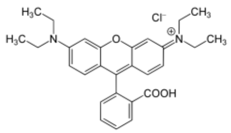 | 479.0 | 15 g L$^{-1}$ (20 ºC) | 1.9-2.0 | - | 887 mg kg$^{-1}$ (Rat, oral) | Positive |
| Acid Orange 7 (AO7) | | 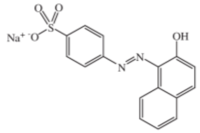 | 350.3 | 116 g L$^{-1}$ (30 ºC) | - | 11.4 | 150 mg kg$^{-1}$ (Rat, oral) | Negative |
| Phenol (PE) | | 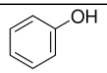 | 94.11 | 50 g L$^{-1}$ (20 ºC) | - | 9.89 | 320 mg kg$^{-1}$ (Rat, oral) | Negative |
| Bisphenol A (BPA) | | 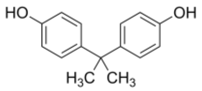 | 228.3 | 0.12 g L$^{-1}$ (20 ºC) | - | 10.29 | - | Negative |
| p-Nitrophenol (PNP) | Phenol derivatives | 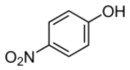 | 139.1 | 16 g L$^{-1}$ (25 ºC) | - | 7.15 | 202 mg kg$^{-1}$ (Rat, oral) | Negative |
| 4-Hydroxybenzoic acid (HBAc) | | 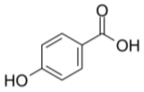 | 138.1 | 5 g L$^{-1}$ (25 ºC) | - | 4.54 | 2200 mg kg$^{-1}$ (Rat, oral) | Negative |
| Ciprofloxacin (CIP) | | 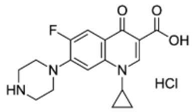 | 331.3 | 86 g L$^{-1}$ (25 ºC) | - | 4.04 | 5000 mg L$^{-1}$ (Rat, oral) | - |
| Tetracycline (TC) | Antibiotic | 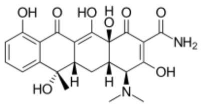 | 480.9 | 50 g L$^{-1}$ (20 ºC) | -1.25 | 3.2 | - | - |

**Supplementary Table 6 | Brief summary of recent catalysts for AOP.**

| Type | Catalyst | Additional oxidant | Contaminant | Dosage of catalyst | Surface area ($m^2\ g^{-1}$) | Time | $k$ ($min^{-1}$) | Metal loading (wt%) | pH | Ref. |
|---|---|---|---|---|---|---|---|---|---|---|
| Pristine MOFs | ZIF-8 | sonolysis/PDS (0.6 mM) | AB7 (10 ppm) | 0.6 g $L^{-1}$ | 1484 | 60 min | 0.028 | 2<br>8.5 | 5.0 | Sisi *et al.*[54] |
| | ZIF-67 | PMS (150 ppm) | RhB (50 ppm) | 0.05 g $L^{-1}$ | 1586 | 60 min | 0.793 | 26.4 | 7.0 | Lin *et al.*[55] |
| | MIL-88A(Fe) | PS (32 mM) | OG (0.2mM) | 1.0 g $L^{-1}$ | 89 | 120 min | ~0.01 | 13.5 | 7.0 | Pu *et al.*[56] |
| | MIL-101(Fe) | UV/PS (500 ppm) | TCEP (3.51 μM) | 0.5 g $L^{-1}$ | - | 180 min | 0.021 | 23.6 | 7.0 | Hu *et al.*[57] |
| MOF composites | $Co_3O_4$@MOFs | PMS (0.8 mM) | 4-CP (0.78 mM) | 0.5 g $L^{-1}$ | 196 | 60 min | - | ~67 | 7.0 | Zeng *et al.*[58] |
| | $Mn_3O_4$@ZIF-8 | PMS (0.3 g $L^{-1}$) | RhB (10 ppm) | 0.4 g $L^{-1}$ | - | 60 min | 0.152 | 24.1 | 5.2 | Hu *et al.*[59] |
| | PBA@PmPDs | PMS (0.2 g $L^{-1}$) | RhB (15 ppm) | 0.1 g $L^{-1}$ | - | 60 min | 0.042 | - | 7.0 | Zeng *et al.*[60] |
| Carbon materials | N-doped graphene (N-G) | PMS (3.25 mM) | PHBA (20 ppm) | 0.1 g $L^{-1}$ | 324 | 30 min | - | 0 | - | Liang *et al.*[61] |
| | NPCs | PMS (1.6 mM) | Phenol (20 ppm) | 0.2 g $L^{-1}$ | 998 | 60 min | 0.079 | 0 | 7.0 | Wang *et al.*[19] |
| | S-nanodiamonds (ND) | PMS (2 g $L^{-1}$) | Phenol (20 ppm) | 0.1 g $L^{-1}$ | 394 | 120 min | 0.030 | 0 | 7.0 | Duan *et al.*[31] |
| | N-SCNTs | PMS (6.5 mM) | Phenol (20 ppm) | 0.1 g $L^{-1}$ | - | 30 min | 0.247 | 0 | 7.0 | Duan *et al.*[18] |
| | commercial CNTs | PDS (1 mM) | Phenol (9.4 ppm) | 0.1 g $L^{-1}$ | - | 60 min | 0.038 | 0 | 7.3 | Ren *et al.*[62] |
| | Zn-Co-ZIFs derived NPCs | PMS (1 g $L^{-1}$) | PHBA (20 ppm) | 0.1 g $L^{-1}$ | 388 | 20 min | 0.547 | 0 | - | Liang *et al.*[63] |
| Carbon composites | ZIF-NC/g-$C_3N_4$ | photo-PMS (2 mM) | BPA (20 ppm) | - | - | 60 min | 0.051 | 0 | - | Gong *et al.*[64] |
| | $Fe_3C$@NSCNTs | PDS (1 mM) | BPA (20 ppm) | 0.2 g $L^{-1}$ | 369 | 20 min | 0.202 | ~10 | 7.0 | Fu *et al.*[65] |
| Metal oxides | Au/$VO_2$ | PDS (15 mM) | RhB (100 ppm) | 0.2 g $L^{-1}$ | - | 1.5 min | 0.176 | 3.7 | - | Xie *et al.*[66] |

| | | | | | | | | | | |
|---|---|---|---|---|---|---|---|---|---|---|
| | $Co_3O_4$-$CeO_2$ | PMS (0.1 mM) | ATZ (0.01 mM) | 5 mg $L^{-1}$ | - | 5 min | 1.21 | 73 | 7.5 | Song *et al.*[67] |
| Metal composites on carbon | MnFeO-CNTs | PMS (1.0 mM) | SMX (10 ppm) | 1.2 g $L^{-1}$ | 103 | 60 min | 0.11 | - | 7.0 | Huang *et al.*[68] |
| | MCFC (Fe-Co oxide) | PMS (0.65 mM) | ABEE (10 ppm) | 10 mg $L^{-1}$ | - | 20 min | 0.647 | 23.7 | 7.0 | Liu *et al.*[69] |
| | Cu-rGO LDH | PMS (3 mM) | BPA (0.09 mM) | 0.25 g $L^{-1}$ | 148 | 40 min | 0.115 | 70 | 7.0 | Shahzad *et al.*[70] |
| Single atom catalysts | $CoN_{2+2}$-C (ZIF-8) | PMS (0.3 g $L^{-1}$) | CIP (20 ppm) | 0.2 g $L^{-1}$ | 1151 | 40 min | 0.14 | - | 7 | Mi *et al.*[71] |
| | FeCo-NC (PBA) | PMS (0.2 g $L^{-1}$) | BPA (20 ppm) | 0.1 g $L^{-1}$ | 375 | 4 min | 1.25 | 20 | 6 | Li *et al.*[24] |
| | $Fe_1$/CN | PMS (1 mM) | 4-CP (50 ppm) | 0.5 g $L^{-1}$ | 196 | 10 min | 1.43 | 11.2 | 6.7 | Zhang *et al.*[23] |
| | Co-$N_3O_1$/TCN | PMS (1 mM) | CIP (5 ppm) | 0.1 g $L^{-1}$ | 83 | 3 min | 0.29 | 10.49 | 6.5 | Wang *et al.*[72] |
| | $Cu_1$/NG | PDS (0.5 m M) | BPA (20 ppm) | 0.1 g $L^{-1}$ | - | 5 min | 1.5 | 5.0 | 7.2 | Wang *et al.*[73] |
| MOF-derived crystalline carbon | MCC-950 | PMS (0.4 g $L^{-1}$) | RhB (20 ppm) | 0.08 g $L^{-1}$ | 890 | 3 s | 57.1 | 5.04 | 6.5 | **This work** |
| | | | AO7 (20 ppm) | | | 5 s | 38.5 | | | |
| | | | MB (20 ppm) | | | 5 s | 47.4 | | | |
| | | | Phenol (20 ppm) | | | 60 s | 5.8 | | | |
| | | | BPA (20 ppm) | | | 90 s | 5.1 | | | |
| | | | PNP (20 ppm) | | | 60 s | 4.7 | | | |
| | | | HBAC (20 ppm) | | | 60 s | 6.7 | | | |
| | | | TC (20 ppm) | | | 90 s | 2.4 | | | |
| | | | CIP (20 ppm) | | | 90 s | 2.6 | | | |

# Supplementary References:


1 Fechler, N., Fellinger, T. P. & Antonietti, M. "Salt templating": a simple and sustainable pathway toward highly porous functional carbons from ionic liquids. *Adv. Mater. Processes* **25**, 75-79 (2013).

2 Huang, L. *et al.* In situ synthesis of ultrathin metal-organic framework nanosheets: a new method for 2D metal-based nanoporous carbon electrocatalysts. *J. Mater. Chem. A* **5**, 18610-18617 (2017).

3 Xuan, C. *et al.* From a ZIF-8 polyhedron to three-dimensional nitrogen doped hierarchical porous carbon: an efficient electrocatalyst for the oxygen reduction reaction. *J. Mater. Chem. A* **6**, 10731-10739 (2018).

4 Qian, Y., An, T., Birgersson, K. E., Liu, Z. & Zhao, D. Web-like interconnected carbon networks from NaCl-assisted pyrolysis of ZIF-8 for highly efficient oxygen reduction catalysis. *Small* **14**, 1704169 (2018).

5 Wang, Q. *et al.* Molten NaCl-assisted synthesis of porous Fe-N-C electrocatalysts with a high density of catalytically accessible $FeN_4$ active sites and outstanding oxygen reduction reaction performance. *Adv. Energy Mater.* **11**, 2100219 (2021).

6 Ding, W. *et al.* Shape fixing via salt recrystallization: a morphology-controlled approach to convert nanostructured polymer to carbon nanomaterial as a highly active catalyst for oxygen reduction reaction. *Journal of the American Chemical Society* **137**, 5414-5420 (2015).

7 Park, K. S. *et al.* Exceptional chemical and thermal stability of zeolitic imidazolate frameworks. *Proceedings of the National Academy of Sciences* **103**, 10186-10191 (2006).

8 Shi, Q., Chen, Z., Song, Z., Li, J. & Dong, J. Synthesis of ZIF-8 and ZIF-67 by steam-assisted conversion and an investigation of their tribological behaviors. *Angewandte Chemie* **123**, 698-701 (2011).

9 Akimbekov, Z. *et al.* Experimental and theoretical evaluation of the stability of true MOF polymorphs explains their mechanochemical interconversions. *Journal of the American Chemical Society* **139**, 7952-7957 (2017).

10 Katsenis, A. D. *et al.* In situ X-ray diffraction monitoring of a mechanochemical reaction reveals a unique topology metal-organic framework. *Nature communications* **6**, 1-8 (2015).

11 Rosen, P. F. *et al.* Heat capacity and thermodynamic functions of crystalline forms of the metal-organic framework zinc 2-methylimidazolate, Zn $(MeIm)_2$. *The Journal of Chemical Thermodynamics* **136**, 160-169 (2019).

12 Zhang, H., Zhao, M., Yang, Y. & Lin, Y. Hydrolysis and condensation of ZIF-8 in water. *Microporous Mesoporous Materials* **288**, 109568 (2019).

13 Deacon, A. *et al.* Understanding the ZIF-L to ZIF-8 transformation from fundamentals to fully costed kilogram-scale production. *Commun Chem* **5**, 18 (2022).

14 Shi, Q., Chen, Z., Song, Z., Li, J. & Dong, J. Synthesis of ZIF-8 and ZIF-67 by steam-assisted conversion and an investigation of their tribological behaviors. *Angew. Chem. Int. Ed.* **50**, 672-675 (2011).

15 Sun, H. *et al.* Reduced graphene oxide for catalytic oxidation of aqueous organic pollutants. *ACS Applied Materials Interfaces* **4**, 5466-5471 (2012).

16 Duan, X. *et al.* Surface-tailored nanodiamonds as excellent metal-free catalysts for organic oxidation. *Carbon* **103**, 404-411 (2016).

17 Shao, P. *et al.* Identification and regulation of active sites on nanodiamonds: establishing a highly efficient catalytic system for oxidation of organic contaminants. *Adv. Funct. Mater.* **28**, 1705295 (2018).

18 Duan, X., Sun, H., Wang, Y., Kang, J. & Wang, S. N-doping-induced nonradical reaction on single-walled carbon nanotubes for catalytic phenol oxidation. *ACS Catalysis* **5**, 553-559 (2015).

19 Wang, G., Chen, S., Quan, X., Yu, H. & Zhang, Y. Enhanced activation of peroxymonosulfate by nitrogen doped porous carbon for effective removal of organic pollutants. *Carbon* **115**, 730-739 (2017).

20 Paraknowitsch, J. P. & Thomas, A. Doping carbons beyond nitrogen: an overview of advanced heteroatom doped carbons with boron, sulphur and phosphorus for energy applications. *Energy Environ. Sci.* **6**, 2839-2855 (2013).

21 Yun, E.-T., Lee, J. H., Kim, J., Park, H.-D. & Lee, J. Identifying the nonradical mechanism in the peroxymonosulfate activation process: singlet oxygenation versus mediated electron transfer. *Environmental Science & Technology* **52**, 7032-7042 (2018).

22 Lee, J., von Gunten, U. & Kim, J. H. Persulfate-Based Advanced Oxidation: Critical Assessment of Opportunities and Roadblocks. *Environ. Sci. Technol.* **54**, 3064-3081, doi:10.1021/acs.est.9b07082 (2020).

23 Zhang, L. S. *et al.* Carbon nitride supported high-loading Fe single-atom catalyst for activation of peroxymonosulfate to generate $^1O_2$ with 100% selectivity. *Angewandte Chemie International Edition* **60**, 21751-21755 (2021).

24 Li, X. *et al.* Single cobalt atoms anchored on porous N-doped graphene with dual reaction sites for efficient Fenton-like catalysis. *Journal of the American Chemical Society* **140**, 12469-12475 (2018).

25 Indrawirawan, S., Sun, H., Duan, X. & Wang, S. Nanocarbons in different structural dimensions (0-3D) for phenol adsorption and metal-free catalytic oxidation. *Applied Catalysis B: Environmental* **179**, 352-362 (2015).

26 Duan, X., Sun, H. & Wang, S. Metal-free carbocatalysis in advanced oxidation reactions. *Acc. Chem. Res.* **51**, 678-687 (2018).

27 Choi, C. H., Park, S. H. & Woo, S. I. Binary and ternary doping of nitrogen, boron, and phosphorus into carbon for enhancing electrochemical oxygen reduction activity. *ACS nano* **6**, 7084-7091 (2012).

28 Duan, X., O'Donnell, K., Sun, H., Wang, Y. & Wang, S. Sulfur and nitrogen co-doped graphene for metal-free catalytic oxidation reactions. *Small* **11**, 3036-3044 (2015).

29 Wang, N. *et al.* Prussian blue analogues derived porous nitrogen-doped carbon microspheres as high-performance metal-free peroxymonosulfate activators for non-radical-dominated degradation of organic pollutants. *J. Mater. Chem. A* **6**, 884-895 (2018).

30 Gao, P. *et al.* Promoted peroxymonosulfate activation into singlet oxygen over perovskite for ofloxacin degradation by controlling the oxygen defect concentration. *Chemical Engineering Journal* **359**, 828-839 (2019).

31 Duan, X. *et al.* Nanodiamonds in $sp^2/sp^3$ configuration for radical to nonradical oxidation: Core-shell layer dependence. *Applied Catalysis B: Environmental* **222**, 176-181 (2018).

32 Lee, H. *et al.* Activation of persulfates by graphitized nanodiamonds for removal of organic compounds. *Environ. Sci. Technol.* **50**, 10134-10142 (2016).

33 Feng, Y., Lee, P.-H., Wu, D. & Shih, K. Surface-bound sulfate radical-dominated degradation of 1, 4-dioxane by alumina-supported palladium ($Pd/Al_2O_3$) catalyzed peroxymonosulfate. *Water Res.* **120**, 12-21 (2017).

34 Xiong, Y. *et al.* Single-atom Fe catalysts for Fenton-like reactions: roles of different N species. *Adv. Mater. Processes* **34**, 2110653 (2022).

35 Zhou, X. *et al.* Identification of Fenton-like active Cu sites by heteroatom modulation of electronic density. *Proceedings of the National Academy of Sciences* **119**, e2119492119 (2022).

36 Yang, J. *et al.* Single Mn atom anchored on N-doped porous carbon as highly efficient Fenton-like catalyst for the degradation of organic contaminants. *Applied Catalysis B: Environmental* **279**, 119363 (2020).

37 Zhao, Z. *et al.* Turning the inert element Zinc into an active single-atom catalyst for efficient Fenton-like chemistry. *Angewandte Chemie International Edition* **62**, e202219178 (2023).

38 Jia, Y. *et al.* Tailoring the electronic structure of an atomically dispersed zinc electrocatalyst: coordination environment regulation for high selectivity oxygen reduction. *Angewandte Chemie International Edition* **61**, e202110838 (2022).

39 Xiao, Y. *et al.* Constructing zinc single-atom catalysts for the direct electron-transfer mechanism in peroxymonosulfate activation to degrade sulfamethoxazole efficiently. *Chemical Engineering Journal* **474**, 145973 (2023).

40 Xin, S. *et al.* Electron delocalization realizes speedy Fenton-like catalysis over a High-loading and low-valence zinc single-atom catalyst. *Advanced science* **10**, 2304088 (2023).

41 Yu, X. *et al.* A green edge-hosted zinc single-site heterogeneous catalyst for superior Fenton-like activity. *Proceedings of the National Academy of Sciences* **120**, e2221228120 (2023).

42 Liu, B., Shioyama, H., Akita, T. & Xu, Q. Metal-organic framework as a template for porous carbon synthesis. *Journal of the American Chemical Society* **130**, 5390-5391 (2008).

43 Jiang, H.-L. *et al.* From metal-organic framework to nanoporous carbon: toward a very high surface area and hydrogen uptake. *Journal of the American Chemical Society* **133**, 11854-11857 (2011).

44 Hu, M. *et al.* Direct carbonization of Al-based porous coordination polymer for synthesis of nanoporous carbon. *Journal of the American Chemical Society* **134**, 2864-2867 (2012).

45 Yang, S. J. *et al.* MOF-derived hierarchically porous carbon with exceptional porosity and hydrogen storage capacity. *Chem. Mater.* **24**, 464-470 (2012).

46 Chaikittisilp, W. *et al.* Nanoporous carbons through direct carbonization of a zeolitic imidazolate framework for supercapacitor electrodes. *Chem. Commun.* **48**, 7259-7261 (2012).

47 Tang, J. *et al.* Thermal conversion of core-hell metal-organic frameworks: a new method for selectively functionalized nanoporous hybrid carbon. *Journal of the American Chemical Society* **137**, 1572-1580 (2015).

48 Pachfule, P., Shinde, D., Majumder, M. & Xu, Q. Fabrication of carbon nanorods and graphene nanoribbons from a metal-organic framework. *Nature chemistry* **8**, 718-724 (2016).

49 Liu, C. *et al.* Hollow mesoporous carbon nanocubes: rigid-interface-induced outward contraction of metal-organic frameworks. *Adv. Funct. Mater.* **28**, 1705253 (2018).

50 Chen, L.-F., Lu, Y., Yu, L. & Lou, X. W. D. Designed formation of hollow particle-based nitrogen-doped carbon nanofibers for high-performance supercapacitors. *Energy Environ. Sci.* **10**, 1777-1783 (2017).

51 Chen, Y. *et al.* Enhanced oxygen reduction with single-atomic-site iron catalysts for a zinc-air battery and hydrogen-air fuel cell. *Nature communications* **9**, 1-12 (2018).

52 Hou, C. C. *et al.* A gas-steamed MOF route to P-doped open carbon cages with enhanced Zn-ion energy storage capability and ultrastability. *Adv. Mater. Processes* **33**, 2101698 (2021).

53 Liu, X. *et al.* Core-shell MOF@ COF motif hybridization: selectively functionalized precursors for titanium dioxide nanoparticle-embedded nitrogen-rich carbon architectures with superior capacitive deionization performance. *Chem. Mater.* **33**, 1657-1666 (2021).

54 Sisi, A. J., Fathinia, M., Khataee, A. & Orooji, Y. Systematic activation of potassium peroxydisulfate with ZIF-8 via sono-assisted catalytic process: mechanism and ecotoxicological analysis. *Journal of Molecular Liquids* **308**, 113018 (2020).

55 Lin, K.-Y. A. & Chang, H.-A. Zeolitic imidazole framework-67 (ZIF-67) as a heterogeneous catalyst to activate peroxymonosulfate for degradation of Rhodamine B in water. *Journal of the Taiwan Institute of Chemical Engineers* **53**, 40-45 (2015).

56 Pu, M. *et al.* Activation performance and mechanism of a novel heterogeneous persulfate catalyst: metal-organic framework MIL-53 (Fe) with Fe II/Fe III mixed-valence coordinatively unsaturated iron center. *Catalysis science technology* **7**, 1129-1140 (2017).

57 Hu, H. *et al.* Enhanced photocatalysis degradation of organophosphorus flame retardant using MIL-101 (Fe)/persulfate: effect of irradiation wavelength and real water matrixes. *Chemical Engineering Journal* **368**, 273-284 (2019).

58 Zeng, T., Zhang, X., Wang, S., Niu, H. & Cai, Y. Spatial confinement of a $Co_3O_4$ catalyst in hollow metal-organic frameworks as a nanoreactor for improved degradation of organic pollutants. *Environmental Science & Technology* **49**, 2350-2357 (2015).

59 Hu, L., Deng, G., Lu, W., Lu, Y. & Zhang, Y. Peroxymonosulfate activation by $Mn_3O_4$/metal-organic framework for degradation of refractory aqueous organic pollutant rhodamine B. *Chinese Journal of Catalysis* **38**, 1360-1372 (2017).

60 Zeng, L. *et al.* Core-shell prussian blue analogues@ poly (m-phenylenediamine) as efficient peroxymonosulfate activators for degradation of Rhodamine B with reduced metal leaching. *Journal of colloid interface science* **534**, 586-594 (2019).

61 Liang, P. *et al.* N-doped graphene from metal-organic frameworks for catalytic oxidation of p-hydroxylbenzoic acid: N-functionality and mechanism. *ACS Sustainable Chemistry & Engineering* **5**, 2693-2701 (2017).

62 Ren, W. *et al.* Activation of peroxydisulfate on carbon nanotubes: electron-transfer mechanism. *Environmental Science & Technology* **53**, 14595-14603 (2019).

63 Liang, P. *et al.* Dual-metal zeolitic imidazolate frameworks and their derived nanoporous carbons for multiple environmental and electrochemical applications. *Chemical Engineering Journal* **351**, 641-649 (2018).

64 Gong, Y. *et al.* MOF-derived nitrogen doped carbon modified g-$C_3N_4$ heterostructure composite with enhanced photocatalytic activity for bisphenol A degradation with peroxymonosulfate under visible light irradiation. *Applied Catalysis B: Environmental* **233**, 35-45 (2018).

65 Fu, H. *et al.* Transformation to nonradical pathway for the activation of peroxydisulfate after doping S into $Fe_3C$-encapsulated N/S-codoped carbon nanotubes. *Chemical Engineering Journal* **409**, 128201 (2021).

66 Xie, M. *et al.* Tailoring the Electronic Metal–Support Interactions in Supported Atomically Dispersed Gold Catalysts for Efficient Fenton-like Reaction. *Angewandte Chemie* **133**, 14491-14496 (2021).

67 Song, C. *et al.* Overturned loading of inert $CeO_2$ to active $Co_3O_4$ for unusually improved catalytic activity in Fenton-like reactions. *Angewandte Chemie* **134**, e202200406 (2022).

68 Huang, M. *et al.* Strong metal-support interaction between carbon nanotubes and Mn-Fe spinel oxide in boosting peroxymonosulfate activation: Underneath mechanisms and application. *Chemical Engineering Journal* **429**, 132372 (2022).

69 Liu, C. *et al.* Novel carbon based Fe-Co oxides derived from prussian blue analogues activating peroxymonosulfate: Refractory drugs degradation without metal leaching. *Chemical Engineering Journal* **379**, 122274 (2020).

70 Shahzad, A. *et al.* Non-radical PMS activation by the nanohybrid material with periodic confinement of reduced graphene oxide (rGO) and Cu hydroxides. *J. Hazard. Mater.* **392**, 122316 (2020).

71 Mi, X. *et al.* Almost 100% peroxymonosulfate conversion to singlet oxygen on single-atom $CoN_{2+2}$ sites. *Angewandte Chemie* **133**, 4638-4643 (2021).

72 Wang, Z. *et al.* Cobalt single atoms anchored on oxygen-doped tubular carbon nitride for efficient peroxymonosulfate activation: simultaneous coordination structure and morphology modulation. *Angewandte Chemie* **134**, e202202338 (2022).

73 Wang, B. *et al.* A site distance effect induced by reactant molecule matchup in single-atom catalysts for Fenton-like reactions. *Angewandte Chemie* **134**, e202207268 (2022).
